# The role of guanidine hydrochloride in graphitic carbon nitride synthesis

**DOI:** 10.1038/s41598-021-01009-8

**Published:** 2021-11-03

**Authors:** Aneta Smýkalová, Kryštof Foniok, Daniel Cvejn, Kamil Maciej Górecki, Petr Praus

**Affiliations:** 1 Department of Chemistry, VSB-Technical University of Ostrava, 17. listopadu 15, 708 00 Ostrava-Poruba, Czech Republic; 2 Institute of Environmental Technology, CEET, VSB-Technical University of Ostrava, 17. listopadu 15, 708 00 Ostrava-Poruba, Czech Republic; 3 ENET Centre, CEET, VSB-Technical University of Ostrava, 17. listopadu 15, 708 00 Ostrava-Poruba, Czech Republic

**Keywords:** Nanoscale materials, Catalyst synthesis, Nanoparticle synthesis, Photocatalysis

## Abstract

Graphitic carbon nitride (CN) was synthesized from guanidine hydrochloride (G), melamine (M) and dicyandiamide (DCDA). The CN materials synthetized from the pure precursors and their mixtures were characterized by common methods, including thermal analysis, and their photocatalytic activities were tested by the degradation of selected organic pollutants, such as amoxicillin, phenol, Rhodamine B (RhB). Remarkable changes in their texture properties in terms of particle sizes, specific surface areas (SSA) and consequently their photocatalytic activity were explained by the role of guanidine hydrochloride in their synthesis. The SSA increased due to the release of NH_3_ and HCl and its complex reactions with melamine and DCDA forming structure imperfections and disruptions. The photocatalytic activity of the CN materials was found to be dependent on their SSA.

## Introduction

Graphitic carbon nitride is a metal-free polymeric semiconductor which has been attracting the attention of scientists for the last decade because of its ability to absorb visible light (band gap energy about 2.7 eV), high thermal and chemical stability, low cost synthesis, etc. The history, properties and possible applications of this material have been already described in many comprehensive review papers, for instance in Refs.^1–7^. The majority of its applications were focused on heterogenous photocatalysis but it is also used for solar cells fabrication^8^, imaging, biotherapy, and the sensing of some compounds^9–12^. Besides physical and chemical vapour deposition, CN has been mostly prepared by the heating of various nitrogen-rich organic precursors, such as melamine^13^, urea^14^, thiourea^15^, triazines^16^, cyanamide^17^, dicyandiamide^18^, cyanuric chloride^19^ and guanidine hydrochloride^20^ or thiocyanate^21^. The chemical synthesis is based on the polycondensation of melamine into melem/melam, melon and finally a polymeric network of carbon and nitrogen^7,22,23^. The other precursors form melamine which further polymerizes to CN through the aforementioned route.

In this work, the synthesis of CN based on the polymerization of guanidine hydrochloride with melamine and guanidine hydrochloride with dicyandiamide has not been published yet. The mixture of guanidine hydrochloride and melamine was found to provide CN with the highest specific surface area (without further exfoliation) and consequently the highest photocatalytic activity. The photocatalytic activity was tested using three different kinds of organic compounds representing various environmental pollutants: amoxicillin, Rhodamine B and phenol.

## Materials and methods

### Chemicals

All chemicals used for the synthesis of all materials, photocatalytic degradation and the determination of phenol were of analytical-reagent grade (pro analysis) and used as obtained. Melamine, dicyandiamide, guanidine hydrochloride, 4-nitroaniline and amoxicillin were purchased from Sigma-Aldrich (Darmstadt, Germany). Phenol and Rhodamine B were obtained from Thermo Fisher Scientific (Waltham, MA, USA). Sodium carbonate, sodium nitrate, ethylenediaminetetraacetic acid (EDTA), p-benzoquinone and t-butanol were purchased from Penta (Chrudim, Czech Republic). Distilled water was used for the preparation of solutions and experiments.

### Synthesis of CN materials

The CN materials were synthetized by a facile method of the direct heating of melamine, dicyandiamide and guanidine hydrochloride or their mixtures at 550 °C for 4 h with a heating rate of 3 °C min^−1^. Typically, 5 g of individual precursors or their mixtures of a particular mass ratio, which were manually mixed in an agate mortar, were placed in a ceramic crucible with a lid in a muffle furnace. The crucible was then cooled down to room temperature out of furnace and ground in a laboratory mill into a fine powder.

The CN materials from the mixtures of guanidine hydrochloride, melamine and dicyandiamide were labelled CN-GM Y and CN-GD Y respectively, where Y is a number and represents the mass ratio of guanidine and the other precursor (melamine or DCDA). The synthetized CN materials used for comparison were labelled as CN-M (CN prepared solely from melamine), CN-D (CN prepared solely from dicyandiamide) and CN-G (CN prepared solely from guanidine hydrochloride). The theoretical composition of the materials is summarized in Table 1.

**Table 1:** Mass and molar ratio of CN materials.

Material	Mass ratio G:X*	Molar ratio G:X*
CN-G	–	–
CN-M	–	–
CN-D	–	–
CN-GM 0.5	0.5:1	0.4:1
CN-GM 1	1:1	0.7:1
CN-GM 2	2:1	1.5:1
CN-GM 3	3:1	2.2:1
CN-GM 4	4:1	3.0:1
CN-GD 0.5	0.5:1	0.6:1
CN-GD 1	1:1	1.1:1
CN-GD 2	2:1	2.2:1
CN-GD 3	3:1	3.3:1
CN-GD 4	4:1	4.4:1

*X stands for melamine (M) and dicyandiamide (D).

#### UV–Vis DR spectrometry

The UV–Vis diffuse reflectance spectra (DRS) were recorded using a Shimadzu UV-2600 spectrophotometer (IRS-2600Plus, Shimadzu, Kyoto, Japan) at room temperature. The diffuse reflectance data obtained were transformed using the Kubelka–Munk equation^24^ as follows

1 $$F{(R}_{\infty })=\frac{{\left(1-{R}_{\infty }\right)}^{2}}{{2R}_{\infty }},$$

where R_∞_ is the diffuse reflectance from a semi-infinite layer.

### X-ray diffraction analysis

The X-ray diffraction (XRD) patterns were recorded using a Rigaku SmartLab diffractometer (Rigaku, Tokyo, Japan) equipped with a detector D/teX Ultra 250. A Co tube (CoKα, λ_1_ = 0.178892 nm, λ_2_ = 0.179278 nm) operated at 40 kV and 40 mA was used as a source of X-ray irradiation. The patterns were recorded between 5° and 90° of 2θ with a step size of 0.01° and speed of 0.5 deg min^−1^. The crystallite size (L) was calculated using Scherrer’s equation^25^ for broadening B(2*θ*) in radians at a half maximum intensity (FWHM) of a diffraction band as

2 $$B\left(2\theta \right)=\frac{K\lambda }{L\cos\theta },$$

where θ is Bragg’s angle, λ is the wavelength of X-rays and K is a constant equal to 0.94 for cubic and 0.89 for spherical crystallites. In this work K = 0.90.

### Fourier transform infrared spectroscopy

The Fourier transform infrared (FTIR) spectra were recorded using a Nicolet iS50 device (Thermo Fisher Scientific, Waltham, MA, USA). A small amount, approximately 200 mg, of the CN material was mixed and homogenized with KBr and pressed to obtain a transparent tablet. Each spectrum consisted of 64 scans at a minimum.

### Specific surface area measurement

The specific surface area of the synthetized materials was determined by the adsorption and desorption of nitrogen at 77 K after sample degassing at room temperature for 24 h under less than 1 Pa vacuum. The SSA was evaluated by means of the Brunauer–Emmett–Teller (BET) theory for the p/p^0^ = 0.05–0.25. For this purpose, a device SORPTOMATIC 1990 series (Thermo Fisher Scientific, Waltham, MA, USA) was employed. The mesopore volumes were calculated based on the Barrett, Joyner and Halenda (BJH) theory.

### Elemental analysis

The elemental composition of the synthetized materials was determined by a Flash 2000 Elemental analyser (Thermo Fisher Scientific, Waltham, MA, USA). The content of carbon, nitrogen and hydrogen was measured, and the content of oxygen was calculated as a difference of 100%. The chlorine content in the CN materials was determined by an X-ray fluorescence spectrometer (XRF) SPECTRO Xepos (SPECTRO Analytical Instruments GmbH, Kleve, Germany).

### Photocatalytic degradation

The photocatalytic activity of the CN materials was tested using Rhodamine B, phenol and amoxicillin in the concentrations of 10 mg L^−1^, 30 mg L^−1^ and 20 mg L^−1^, respectively. Each suspension prepared for the photocatalytic degradation contained 45 mg of the CN material and 150 mL of the model pollutant solution. Before the photocatalytic degradation, each mixture was stirred in a glass cylindrical vessel of 200 mL with 80 mm in height and a diameter of 57 mm in the dark for 60 min to reach adsorption–desorption equilibrium and then was irradiated from the top with an LED source (420 nm, intensity of 7.1 mW cm^−2^) for 120 min; the reaction suspension temperatures were kept at 20 °C. The aliquots of 2 mL were taken at regular intervals and filtered using syringe filters Chromafil GF/RC-20/25 (the pore size of 0.2–1 µm).

The photocatalytic activity tested on Rhodamine B was evaluated by measuring the absorbance at 554 nm using a Helios Alpha spectrometer (Thermo Fisher Scientific, Waltham, MA, USA). The amount of decomposed amoxicillin was determined using a high-performance liquid chromatograph (HPLC) Waters 2996 (Waters Corporation Milford, MA, USA) with a PDA detector. For the HPLC separation a Synergi 4 µm Polar-RP 80 Å (100 × 3 mm) column was used. The mobile phase consisted of the methanol solution of 5 mmol L^−1^ ammonium formate and the water solution of 5 mmol L^−1^ ammonium formate (30:70, v/v) with the 0.5 mL min^−1^ flow.

For the determination of phenol 1 mL of the decomposed phenol solution and 9 mL of distilled water were put in a beaker, 4 mL of 5% solution of sodium carbonate was added and mixed. Then, 4 mL of a diazotized solution of 4-nitroaniline was added, mixed and after 15 min the absorbance was measured at 470 nm. The diazotized colourless solution of 4-nitroaniline was prepared by adding 8–10 drops of the saturated sodium nitrate solution to a 40 mL of 5 mmol L^−1^ 4-nitroaniline dissolved in the diluted HCl solution (1:9).

#### Application of scavengers

In a typical experiment with scavengers, the initial concentration of Rhodamine B and the photocatalysts were identical to the photocatalytic activity test. EDTA was used as the scavenger of the holes, p-benzoquinone as the scavenger of superoxide radicals and terc-butanol as the scavenger of hydroxyl radicals. The concentration of every scavenger in a storage bottle was 1 mmol L^−1^. An aliquot of 25 mL for every scavenger was added to 125 mL of the catalyst suspension and Rhodamine B.

### Photoluminescence spectroscopy

Steady-state and time-resolved photoluminescence (PL) measurements were carried out using a FLS980 fluorescence spectrometer (Edinburgh Instruments, UK) equipped with a 450 W xenon arc lamp and an EPL-375 ps pulsed diode laser (λ_em_ = 372 nm with a pulse width of 66.5 ps, a repetition rate of 10 MHz and an average power of 75 µW (Edinburgh Instruments, UK) as excitation sources. PL decay curves were fitted using a multi-exponential function:

3 $$I\left(t\right)=\sum_{i=1}^{3}{B}_{i}{e}^{-t/{\tau }_{i}},$$

where I(t) is the intensity of photoluminescence, t is the time, B_*i*_ coefficients are the time-invariant constants and τ_i_ are the decay times (decay constants). A mean decay time τ_m_ was calculated as

4 $${\tau }_{m}=\frac{{B}_{1}{\tau }_{1}^{2}+{B}_{2}{\tau }_{2}^{2}+{B}_{3}{\tau }_{3}^{2}}{{B}_{1}{\tau }_{1}+{B}_{2}{\tau }_{2}+{B}_{3}{\tau }_{3}}.$$

### Scanning electron microscopy

For the microscopic investigations of the synthetized materials a scanning electron microscope Tescan Vega (Tescan Orsay Holding, Brno, Czech Republic) with a tungsten cathode and an energy-dispersive X-ray spectrometer (EDAX, Ametex, PA, USA) was used. Micrographs were obtained using a mix of the signals of secondary electrons (SE) and backscattered electrons (BSE) mode to get the benefit of both techniques (SE + BSE) while reducing the impact of their drawbacks. The particles sized were evaluated from the SEM micrographs by means of the free software Image J (National Institutes of Health, Maryland, USA).

### Thermogravimetric analysis with differential scanning calorimetry

A device NETZSCH STA 449 F3 Jupiter with an S-type measurement holder was use for thermogravimetric analysis (TGA) simultaneously with differential scanning calorimetry (DSC). Approximately 10 mg of the precursors and their mixtures was put into an Al_2_O_3_ crucible without a lid. Before their heating, the inner space of a furnace was flushed at 20 °C with high purity argon with a flow of 240 mL min^−1^ for one hour. A sample of the pure precursor or their mixture was heated up to 900 °C with a linear heating rate of 5 °C min^−1^. The constant argon flow of 70 mL min^−1^ was kept during the whole analysis.

### Statistical data analysis

The statistical data analysis was performed at the α = 0.05 significance level using the software package QC.Expert (Trilobyte, Czech Republic).

## Results and discussion

### UV–Vis spectroscopy

**Figure 1:**
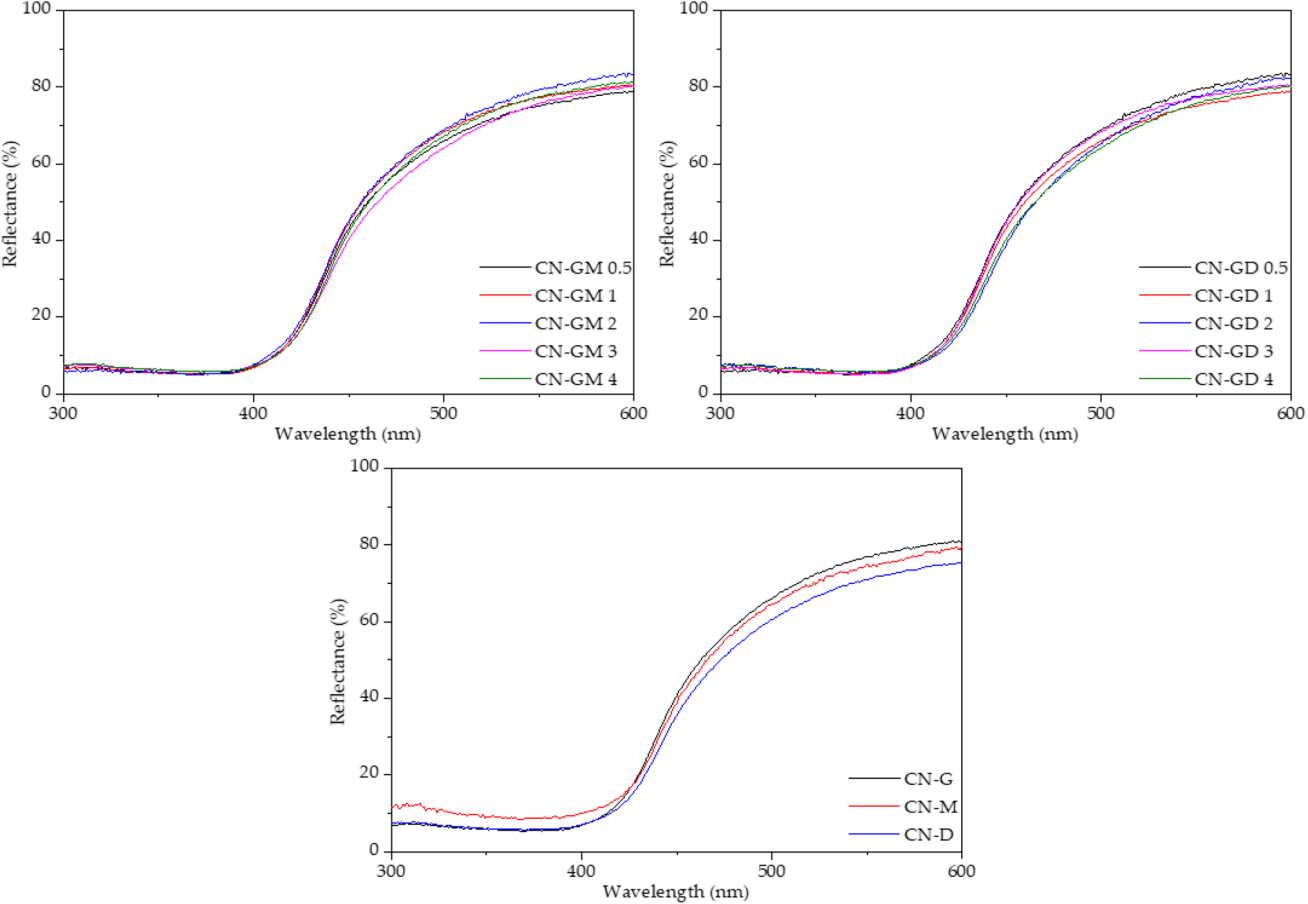
UV–Vis diffuse reflectance spectra of CN materials.

After the synthesis and grounding there were no significant visible differences between the CN materials (see Supplementary materials, Fig. S1). The UV–Vis DRS spectra were recorded to observe light absorption properties (Fig. 1) and mainly to determine the optical band gap energies of the synthetized materials.

The optical band gap (further only band gap) energies (E_g_) listed in Table 2 are randomly changing in the range of 2.67 eV to 2.73 eV. These values (n = 13) were statistically tested: their normality was confirmed (skewness = − 0.701, kurtosis = 3.05, p = 0.454 for the moment test, p = 0.184 for the Kolmogorov–Smirnov test, p = 0.458 for the D’Agostino test). The band-gap energies were determined using the well-known Tauc method^26^ as follows

5 $$\varepsilon h\nu ={C(h\nu -{E}_{g})}^{p},$$

where *ε* is the molar extinction coefficient, *hν* is the energy of incident photons, *C* is a constant and *p* is power depending on the type of electron transition. The power *p* = 2 and *p* = ½ are for direct and indirect semiconductors, respectively. In this work, p = ½ was used^27^. The Tauc plots are shown in Fig. S2.

**Table 2:** Band gap energy, specific surface area and mesopore volume of CN materials.

Material	E_g_ (eV)	SSA (m^2^ g^−1^)	Mesopore volume (cm^3^g^−1^) × 10^–3^
CN-G	2.71	23	9.00
CN-M	2.67	12	5.73
CN-D	2.69	8	2.62
CN-GM 0.5	2.69	25	9.52
CN-GM 1	2.73	29	11.3
CN-GM 2	2.73	54	16.2
CN-GM 3	2.72	23	8.67
CN-GM 4	2.71	23	7.95
CN-GD 0.5	2.71	20	7.95
CN-GD 1	2.72	34	11.8
CN-GD 2	2.71	35	10.7
CN-GD 3	2.70	23	7.35
CN-GD 4	2.71	25	8.47

### Physisorption of nitrogen

The specific surface area of synthetized materials was measured by the physisorption of nitrogen and was evaluated using the BET method, see Table 2. The adsorption–desorption isotherms for all materials are shown in Fig. S3. The hysteresis loops demonstrate the existence of mesopores in these materials.

The SSA of CNs prepared from the single precursor decreased in the sequence CN-G > CN-M > CN-D. The materials synthetized from the mixture of two parts of guanidine hydrochloride and one part of melamine (CN-GM 2) and dicyandiamide (CN-GD 2) had the highest values of SSA within the CN-GM series and CN-GD series, respectively. The material CN-GM 2 had the highest mesopore volume followed by CN-GD 1; CN-GM 1 and CN-GD 2. A strong correlation (r = 0.966) between the SSA and the mesopore volume was found which indicates the dominating mesopore structure of these materials.

### X-ray diffraction analysis

**Figure 2:**
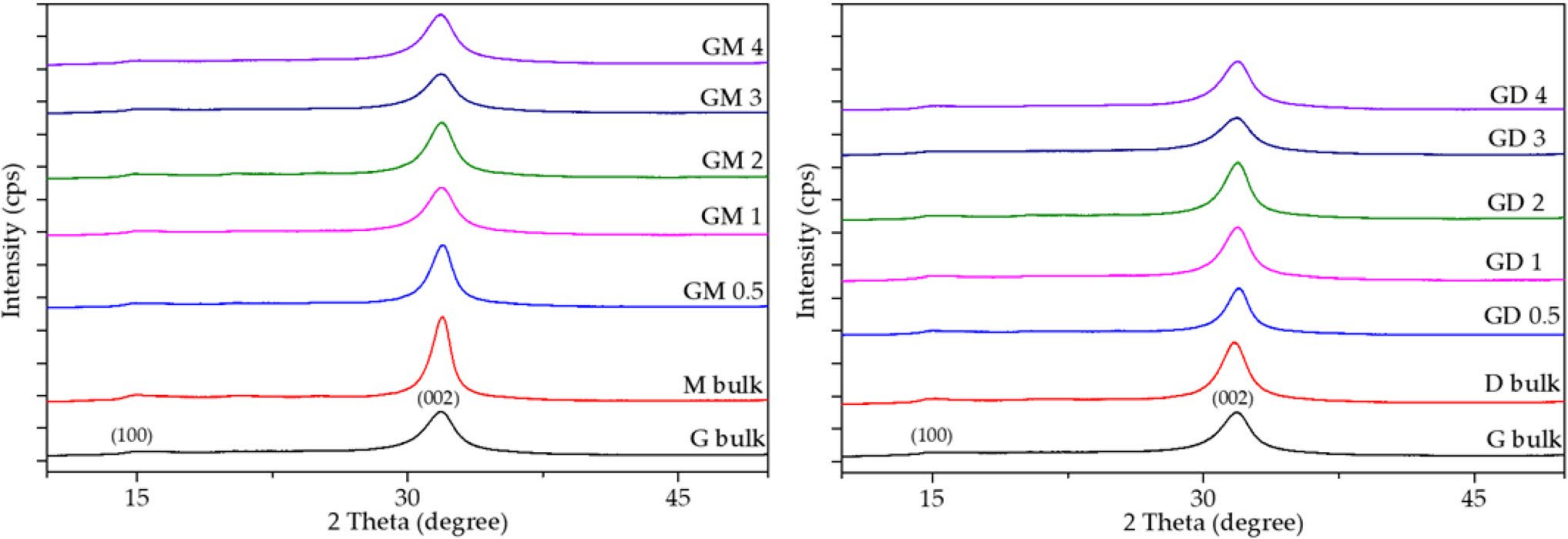
XRD patterns of CN materials (Co K_α_).

The XRD patterns of synthetized CN materials were very similar to CN ones synthetized from single precursors (Fig. 2). The typical graphitic carbon structures with the main diffractions at 31.9° (002) and 14.8° (100) attributed to interlayer stacking of the (002) melem planes and in-plane ordering of the nitrogen-linked heptazine units^28^ were observed.

Roughly speaking, the size of crystallite L(002) calculated from the (002) diffractions diminished with the rising amount of guanidine hydrochloride in the mixtures (Table 3). The d(002) spacings were similar indicating no changes in the CN layer stackings in dependence on the used precursors.

**Table 3:** Selected XRD characteristics of CN materials.

Material	2 Theta (deg)	FWHM (deg)	L(002) (nm)	d(002) (nm)
CN-G	31.82	1.96	4.71	0.326
CN-M	31.93	1.23	7.50	0.325
CN-D	31.77	1.69	5.46	0.327
CN-GM 0.5	31.93	1.43	6.45	0.325
CN-GM 1	31.88	1.88	4.91	0.326
CN-GM 2	31.93	1.82	5.07	0.325
CN-GM 3	31.86	2.02	4.57	0.326
CN-GM 4	31.84	1.98	4.66	0.326
CN-GD 0.5	31.96	1.50	6.15	0.325
CN-GD 1	31.91	1.84	5.01	0.325
CN-GD 2	31.89	1.70	5.43	0.326
CN-GD 3	31.84	2.16	4.27	0.326
CN-GD 4	31.91	1.96	4.71	0.325

### FTIR spectroscopy

**Figure 3:**
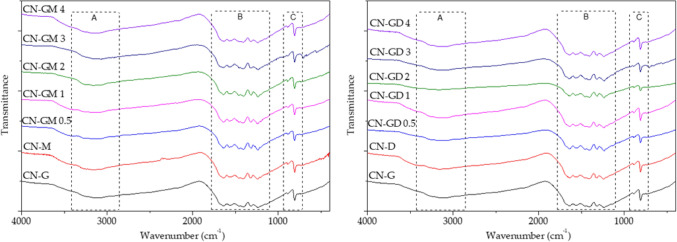
FTIR spectra of CN materials.

The structure of the prepared materials was studied by FTIR spectrometry and their spectra are displayed in Fig. 3. Two regions A and B, which are typical for graphitic carbon nitride, were observed^29–31^. The bands around 3500 cm^−1^ are related to the stretching vibrations of –OH groups. The bands in region A are related with the stretching vibrations of N–H bonds and the bands in region B are related with the stretching vibrations of the C=N and C–N bonds of heterocyclic rings. The breathing mode of triazine units is visible around 810 cm^−1^. The FTIR spectra of all the CN materials were similar and no effect of the used precursors was observed. The small bands around 710 cm^−1^ observed in the CN-GM3 and CN-GD3 were explained by the presence of some laboratory contaminants.

### Elemental analysis

The results of elemental analysis of the CN materials are summarized in Table 4. The C, H and N contents were determined by the elemental analyser and the content of O was calculated up to 100%. The C/N molar ratio was similar (around 0.66) for all the materials which indicates their similar final structures. There were differences in the contents of hydrogen and oxygen. CN-G was more oxidized by air oxygen (4.51 wt%) than CN-M (1.88 wt%) and CN-D (0.87%) likely due to the oxidation tendency of guanidine hydrochloride. In the case of the CN-GM and CN-GD materials (n = 10) the content of oxygen changed randomly from 1.34 to 3.92 wt%. Their normality was confirmed by the Kolmogorov–Smirnov test (p = 0.633), the moment test (p = 0.866) and the D’Agostino test (p = 0.470). The presence of chlorine was not detected by XRF.

**Table 4:** Elemental composition of CN materials.

Material	C (wt%)	H (wt%)	N (wt%)	O (wt%)	C/N molar ratio
CN-G	34.00	1.39	60.10	4.51	0.660
CN-M	34.93	1.72	61.47	1.88	0.663
CN-D	34.70	3.43	61.00	0.87	0.664
CN-GM 0.5	34.73	3.04	60.89	1.34	0.665
CN-GM 1	34.80	1.44	61.50	2.26	0.660
CN-GM 2	34.64	2.62	60.79	1.95	0.665
CN-GM 3	34.41	2.47	60.48	2.64	0.664
CN-GM 4	34.10	1.48	60.50	3.92	0.658
CN-GD 0.5	34.45	2.50	60.56	2.49	0.664
CN-GD 1	34.40	1.68	61.10	2.82	0.657
CN-GD 2	34.52	2.50	60.69	2.29	0.664
CN-GD 3	34.05	2.66	59.81	3.48	0.664
CN-GD 4	34.70	1.51	61.10	2.69	0.663

### Photocatalytic degradation of organic compounds

**Figure 4:**
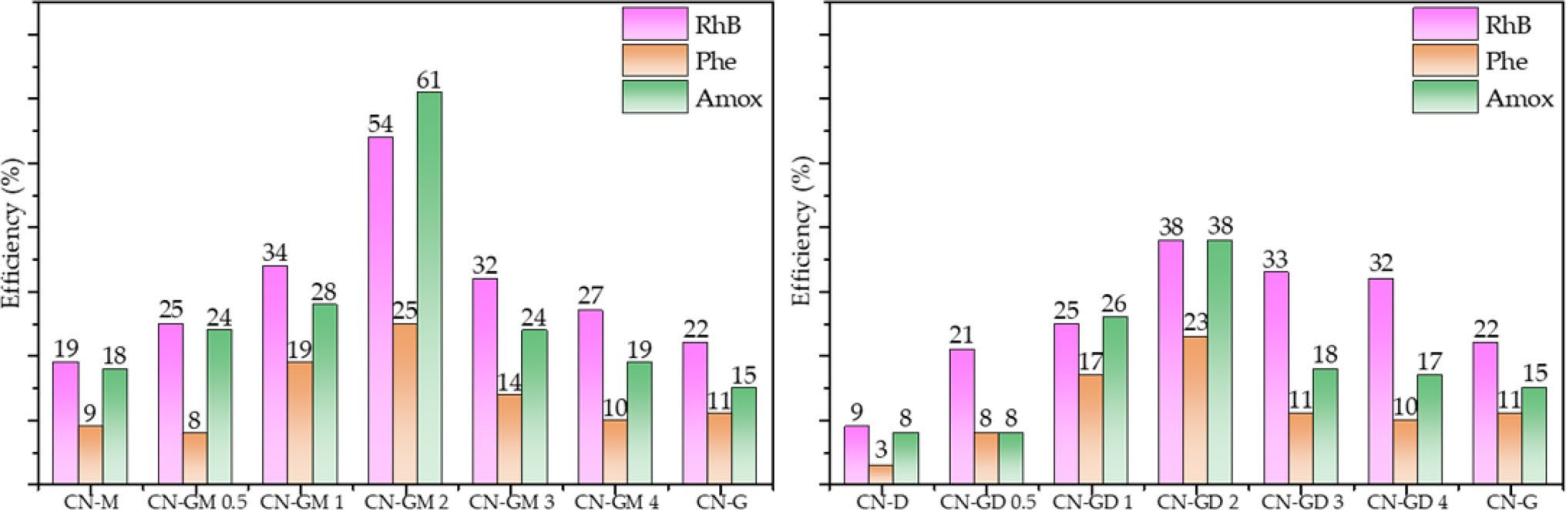
Photocatalytic degradation of RhB, phenol (Phe) and amoxicillin (Amox) after 120 min of visible light irradiation.

**Figure 5:**
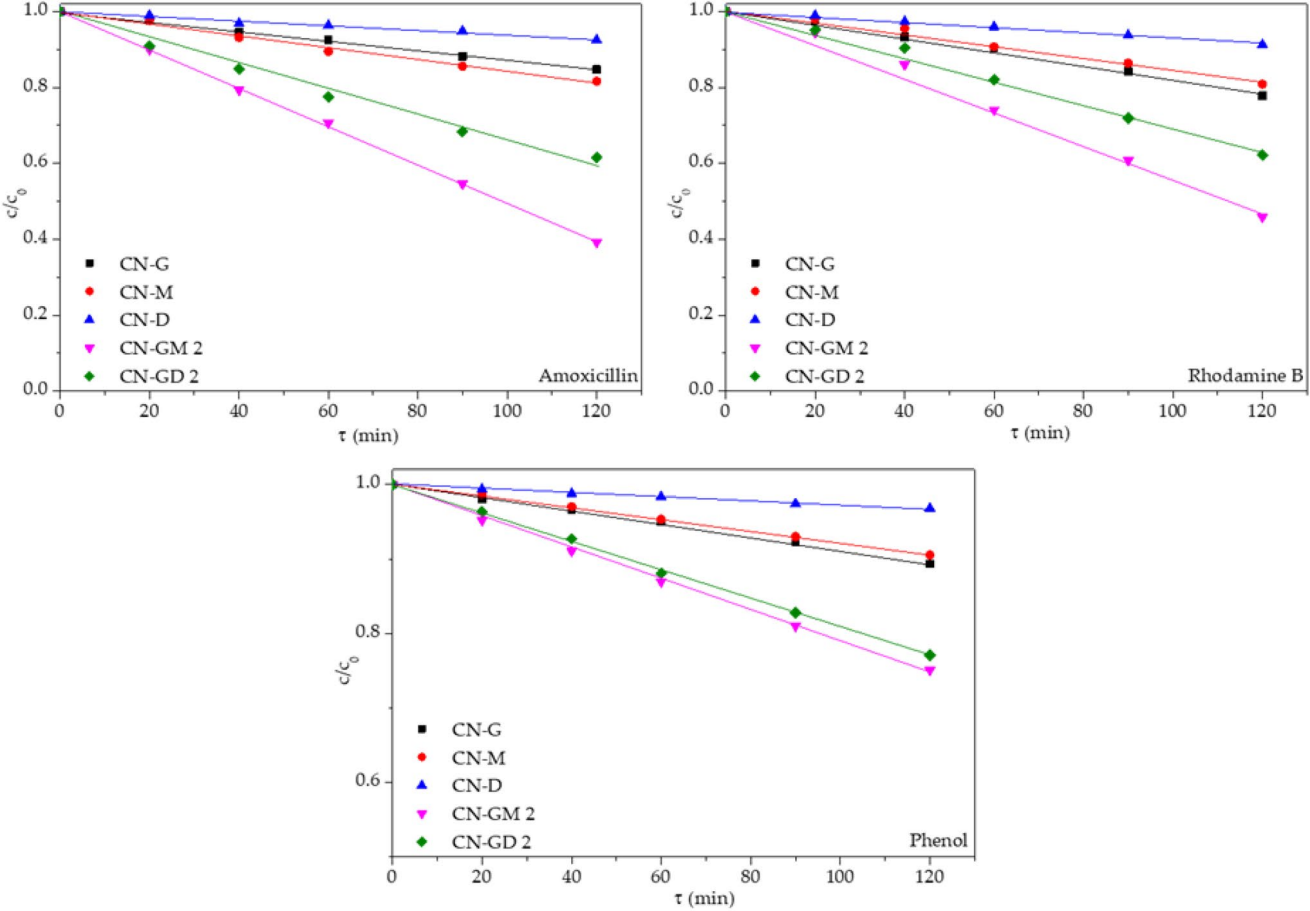
Kinetics curves of photocatalytic degradation of amoxicillin, RhB and phenol (CN-G, CN-M, CN-D, CN-GM 2 and CG-GD 2) (The LED irradiation of 420 nm and the intensity of 7.1 mW cm^−2^). The photocatalysts concentration was 0.3 g L^−1^.

The photocatalytic degradations of RhB, phenol and amoxicillin were performed under the LED irradiation of 420 nm. The photolysis of these compounds was not observed. Figure 4 shows the degradation efficiency for different CN materials after 120 min. The maximal photocatalytic activity of CN-GM 2 and CN-GD 2 can be explained by their maximal specific surface areas. The selected kinetic curves of the photocatalytic degradation of RhB, phenol and amoxicillin are shown in Fig. 5.

The linear relationships of c/c_0_ *versus* time describing the zero-order reactions were found for all the organic compounds and can be derived from the definition of the zero-order reaction rate *r* as

6 $$r=-\frac{dc}{dt}=k,$$

where *k* is the kinetic constant and *c* is the actual concentration of the organic compounds. One can see that the photocatalytic activity decreased in the sequence CN-GM 2 > CN-GD 2 > CN-M ≈ CN-G > CN-D in consistency with their SSA which is in agreement with the results displayed in Fig. 4.

The kinetic constants for all the tested organic compounds and all the CN materials are summarised in Table 5. From this data we can see that (i) CN-GM 2 and CN-GD 2 were the most active photocatalysts (CN-GM 2 was better than CN-GD 2), (ii) the presence of guanidine hydrochloride in the precursor mixtures mostly improved the photocatalytic activities of the final CN materials and (iii) the degradation efficiency of the organic compounds decreased in the sequence RhB > amoxicillin > phenol likely due to their different degradation mechanisms. The photocatalytic degradation of RhB is direct through chromophore cleavage without any visible light absorbing products^37^. The photocatalytic degradation of amoxicillin leads through two pathways^38^ and the complex degradation of phenol leads through catechol, hydroquinone and benzoquinone to various intermediates which are further degraded^39,40^.

**Table 5:** Kinetic constants for the degradation of amoxicillin, RhB and phenol and mean decay times of CN materials.

Material	k(amoxicillin) × 10^–3^ (mol L^−1^ min^−1^)	k(RhB) × 10^–3^ (mol L^−1^ min^−1^)	k(phenol) × 10^–3^ (mol L^−1^ min^−1^)	τ_m_ (ns)
CN-G	1.28 ± 0.08	1.87 ± 0.24	0.871 ± 0.057	9.1
CN-M	1.57 ± 0.21	1.64 ± 0.24	0.795 ± 0.031	8.0
CN-D	0.612 ± 0.075	0.737 ± 0.055	0.267 ± 0.025	8.8
CN-GM 0.5	1.98 ± 0.11	2.15 ± 0.10	0.754 ± 0.123	9.4
CN-GM 1	2.34 ± 0.13	2.87 ± 0.16	1.53 ± 0.16	9.2
CN-GM 2	5.05 ± 0.15	4.63 ± 0.51	2.05 ± 0.09	10.2
CN-GM 3	2.10 ± 0.18	2.68 ± 0.13	1.21 ± 0.08	9.5
CN-GM 4	1.56 ± 0.14	2.22 ± 0.13	0.928 ± 0.115	9.1
CN-GD 0.5	0.994 ± 0.223	1.70 ± 0.06	0.678 ± 0.027	8.9
CN-GD 1	2.20 ± 0.14	2.11 ± 0.11	1.39 ± 0.07	9.1
CN-GD 2	3.54 ± 0.75	3.14 ± 0.09	1.92 ± 0.08	9.3
CN-GD 3	1.55 ± 0.26	2.78 ± 0.10	0.900 ± 0.048	9.9
CN-GD 4	1.47 ± 0.26	2.72 ± 0.07	0.819 ± 0.049	9.4

The statistically significant correlations of the kinetics constants and the SSA, such as r = 0.916 for amoxicillin, r = 0.898 for RhB and r = 0.886 for phenol, indicates the photocatalytic activity depends on the specific surface area of these CN materials. The results obtained in this work were briefly compared with those found in the literature, see Table 6. It is obvious, for example, that the photocatalytic efficiency of 54% for CN-GM 2 is comparable with those of the other authors and is lower than 75% published in the paper^35^. However, in this work a lower loading of the photocatalyst and a shorter irradiation time were used.

**Table 6:** Comparison of obtained results with data published in literatures.

Precursor	Method of synthesis	SSA (m^2^ g^−1^)	Compound, concentration (mg L^−1^)	Concentration of photocatalyst (g L^−1^)	Irradiation time (min)	Efficiency (%)	References
Guanidine hydrocholoride	5 g, calcination in air 550 °C, 3 °C/min, 4 h	23	Rhodamine B 10	0.3	120	22	CN-G (this work)
Melamine	5 g, calcination in air 550 °C, 3 °C/min, 4 h	12	Rhodamine B 10	0.3	120	19	CN-M (this work)
Dicyandiamide	5 g, calcination in air 550 °C, 3 °C/min, 4 h	8	Rhodamine B 10	0.3	120	9	CN-D (this work)
Guanidine hydrochloride and melamine	5 g, calcination in air 550 °C, 3 °C/min, 4 h	54	Rhodamine B 10	0.3	120	54	CN-GM 2 (this work)
Guanidine hydrochloride and dicyandiamide	5 g, calcination in air 550 °C, 3 °C/min, 4 h	35	Rhodamine B 10	0.3	120	38	CN-GD 2 (this work)
Guanidine hydrochloride	4 g, calcination in air 550 °C, 3 °C/min, 3 h	16	Rhodamine B 5	0.5	20	52	^32^
Guanidine carbonate	calcination in air 550 °C, 6 °C/min, 3 h	19	Methyl orange 20	4	120	24	^23^
Dicyandiamide	calcination in air 550 °C; 6 °C/min; 3 h	18	Methyl orange 20	4	120	13	^23^
Melamine	calcination in air 550 °C, 6 °C/min, 3 h	10	Methyl orange 20	4	120	30	^23^
Melamine	calcination in air 550 °C, 3 °C/min; 3 h	7	Methyl orange 20	1	300	24	^33^
Melamine	5 g; calcination in air 560 °C, 4.5 °C/min, 2 h	–	Rhodamine B 10	0.3	60	25	^34^
Dicyandiamide	3 g; calcination in air 550 °C, 4 h	10	Rhodamine B 10	1	180	75	^35^
Dicyandiamide	2 g; calcination in air 550 °C, 5 °C/min, 4 h	14	Rhodamine B 10	1	90	60	^36^

**Figure 6:**
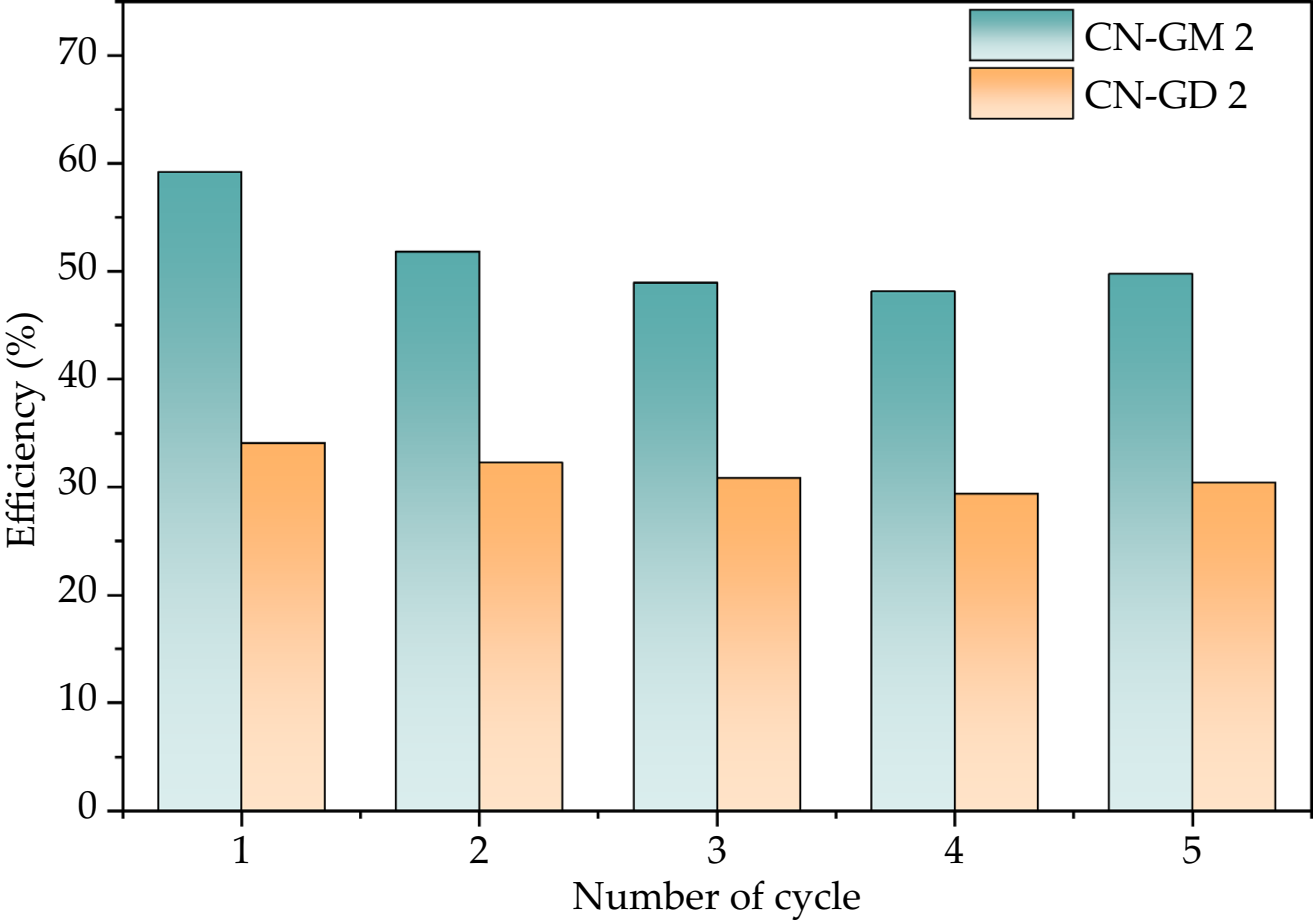
Stability tests of CN-GM 2 and CN-GD 2 materials.

The stability of the CN materials was tested in 5 cycles of the photocatalytic degradation of amoxicillin (Fig. 6). After every cycle the materials were filtered, washed with distilled water and dried at 105 °C until their constant weight. The degradation efficiency of CN-GM 2 decreased more, about 9%, than for CN-GD 2 after the first run. However, the efficiency of both materials stayed nearly constant for the other runs. The first run decrease was likely caused by the loss of small particles during the first filtration. Moreover, the stability was also confirmed by the XRD analysis of the most active materials CN-GD 2 and CN-GM 2 after the fifth run. No structural changes were observed as shown in Fig. S4. The stability tests showed that the CN materials are stable against photocatalytic degradation.

#### Study of photocatalytic mechanisms

**Figure 7:**
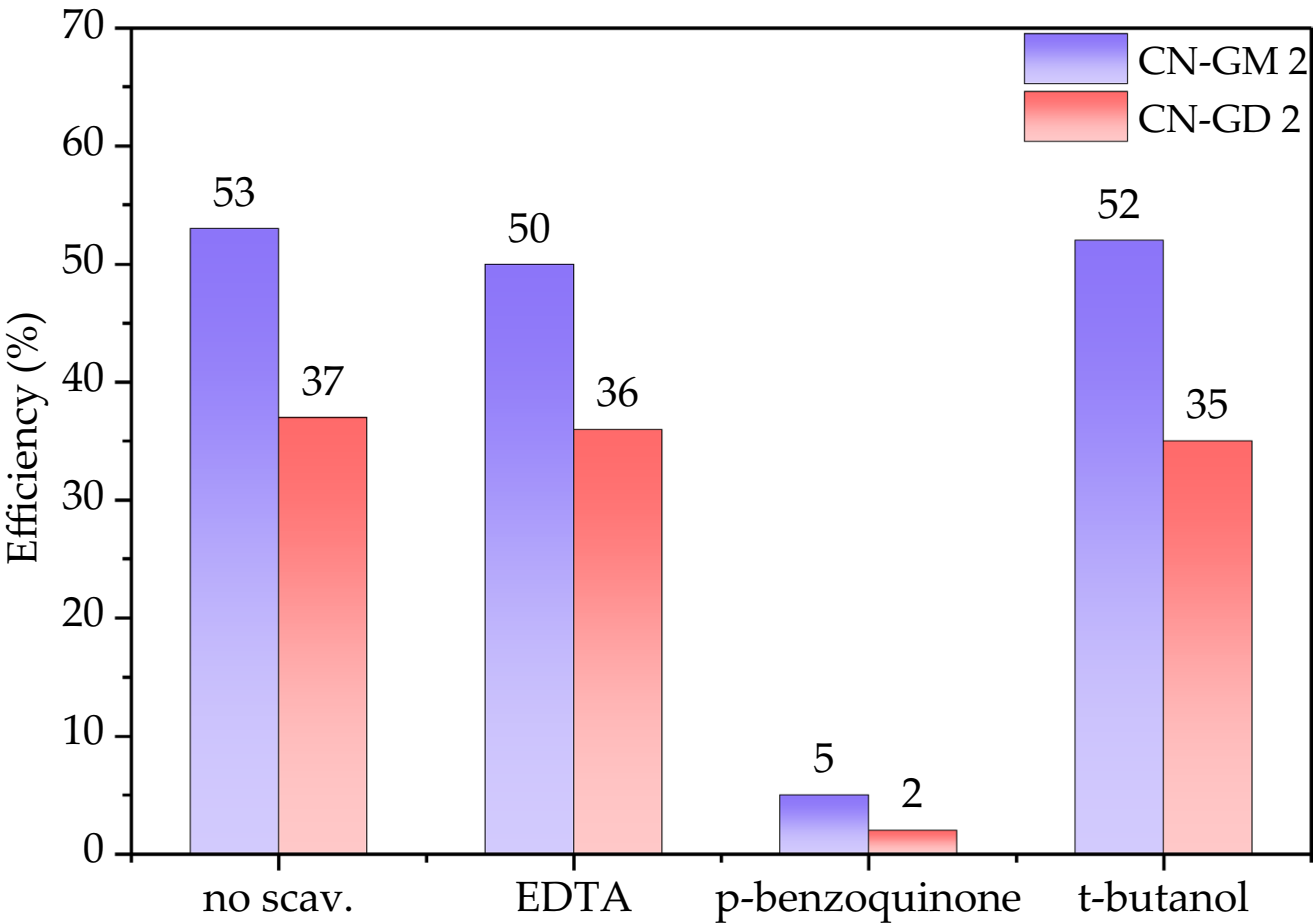
Photocatalytic decomposition of RhB in the presence of CN-GM 2 and CN-GD 2 using scavengers of holes (EDTA), superoxide radicals (p-benzoquinone) and hydroxyl radicals (t-butanol). The decomposition time was 120 min. The RhB concentration was 10 mg L^−1^.

The photocatalytic mechanisms of the photocatalytic degradation were studied using suitable scavengers. Holes, superoxide and hydroxyl radicals were scavenged with EDTA, p-benzoquinone and t-butanol, respectively. The changes in the photocatalytic activity are shown in Fig. 7. It is obvious the photocatalytic efficiency significantly decreased when p-benzoquinone was applied. It indicates that the main photocatalytic agents were superoxide radicals formed according to the reactions

7 $${\text{CN }} + {\text{ h}}\upsilon \, \to {\text{ e}}^{ - } \left( {{\text{CN}}} \right) + {\text{ h}}^{ + } \left( {{\text{CN}}} \right),$$

8 $${\text{e}}^{ - } + {\text{ O}}_{{2}} \to {\text{ O}}_{{2}}^{ \cdot - } ,$$

This finding is consistent with our previous results e.g.^41,42^.

### PL spectroscopy

The lifetimes of photoinduced electrons and holes were measured in order to understand the photocatalytic process. In general, the fast recombination of photoinduced charges is the crucial problem for photocatalysts. Their normalized PL excitation and emission spectra (Fig. S5) showed broad excitation bands around 400 nm and emission bands around 480 nm.

**Figure 8:**
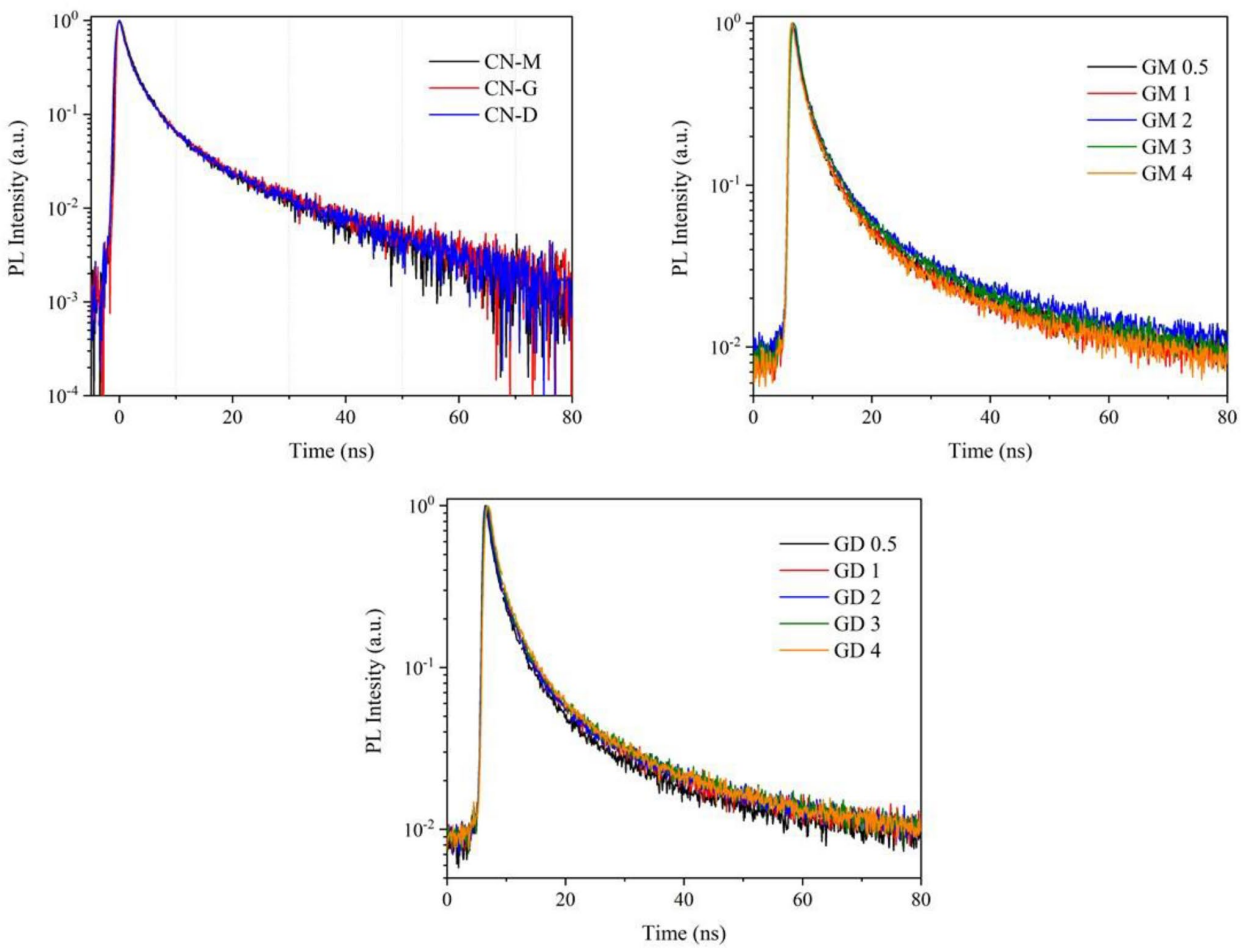
PL decay curves of CN materials.

The PL decay curves were recorded and fitted with exponential relationships to estimate decay times for each CN material, see Fig. 8. The best fit was obtained for the 3rd order kinetic model according to Eq. (3) and mean decay times are summarized in Table 5. The Χ^2^ values are given in Table S1 (Supplementary materials). The B_i_ coefficients and the decay times τ_i_ are given in Table S2. The exponential curves indicate the presence of localised luminescence centres, such as impurities and/or defects, in all the CN materials. This luminescence process is called a monomolecular process and is different from a bimolecular process caused by the recombination of photoinduced electrons and holes^43^. In this case, the decay curves are not exponential but theoretically I(t) ≈ t^−2^.

The statistical analysis of the mean decay times shows that they changed randomly from 8 ns (CN-M) to 10.2 ns (CN-GM 2). The data normality (n = 13) was proved by several tests: skewness = − 0.388, kurtosis = 3.87, p = 0.690 for the moment test, p = 0.497 for the Kolmogorov–Smirnov test, p = 0.671 for the D’Agostino test). From this it follows that no effect of the CN material compositions on their PL decay times was found. Their photocatalytic activity depended on the specific surface area as mentioned above.

### Scanning electron microscopy

**Figure 9:**
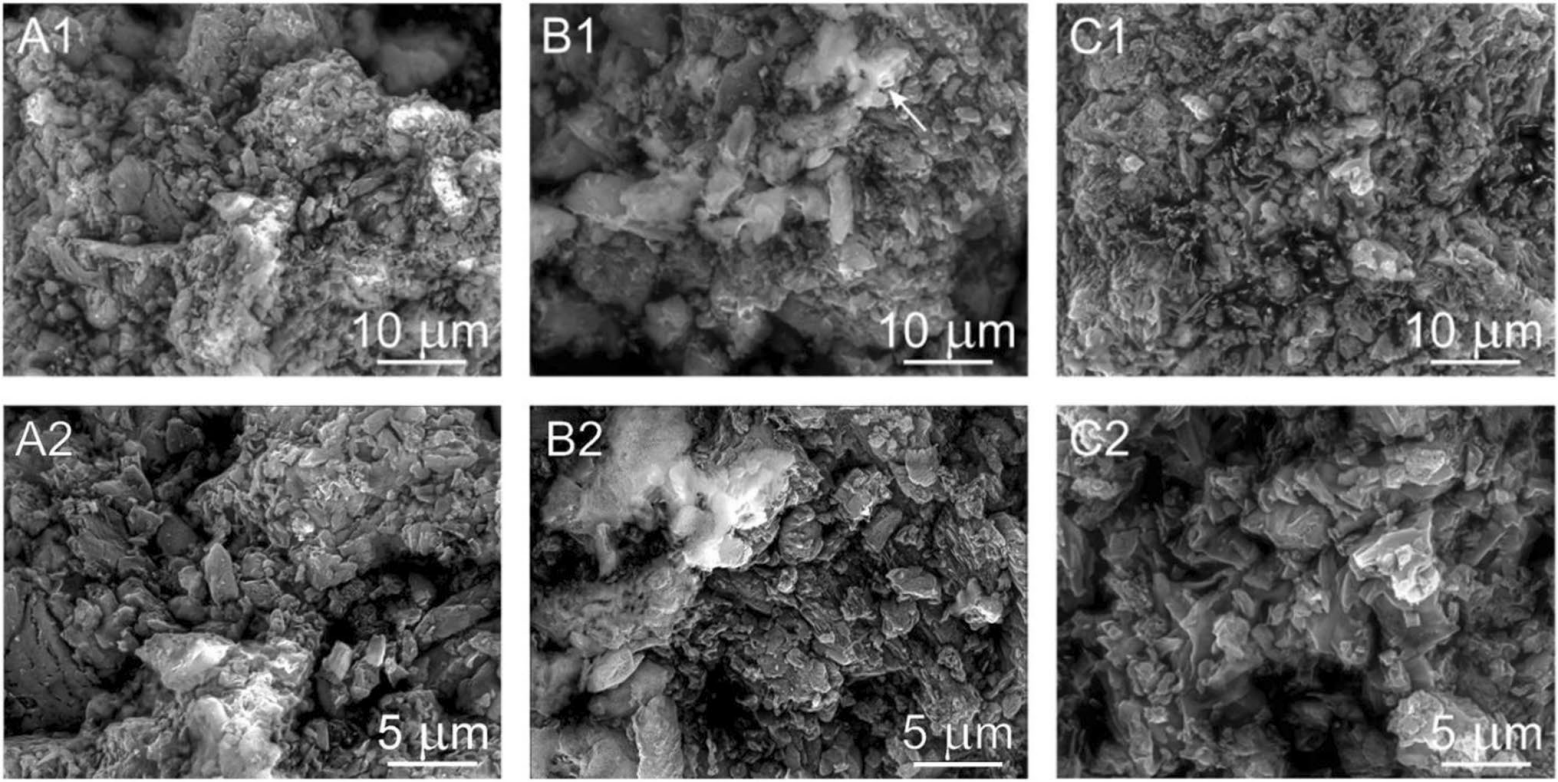
Micrographs (SEM:SE + BSE) of CN-D (**A1,A2**), CN-M (**B1,B2**) and CN-G (**C1,C2**).

**Figure 10:**
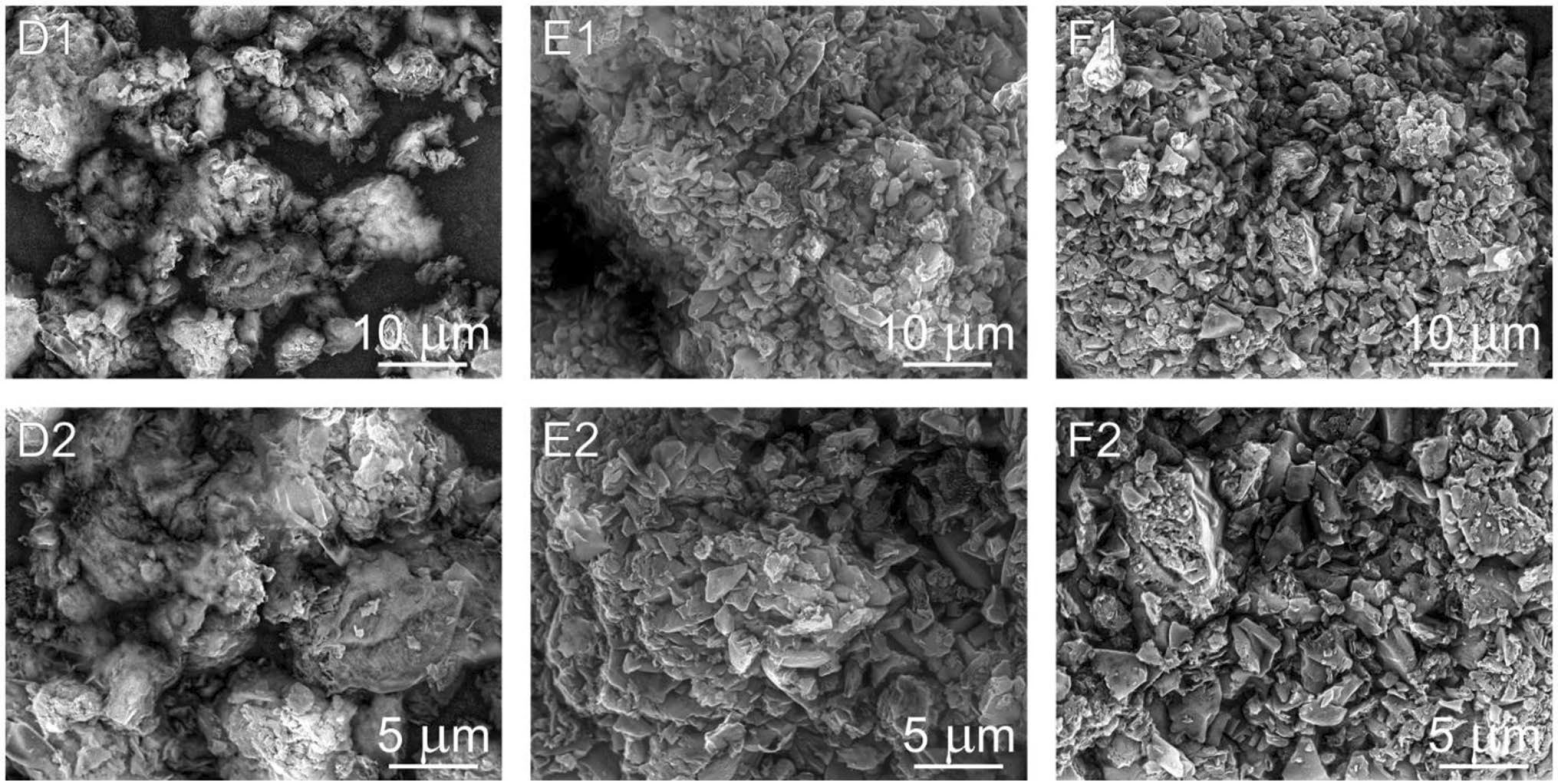
Micrographs (SEM:SE + BSE) of CN-GD 0.5 (**D1,D2**), CN-GD 2 (**E1,E2**) and CN-GD 4 (**F1,F2**).

**Figure 11:**
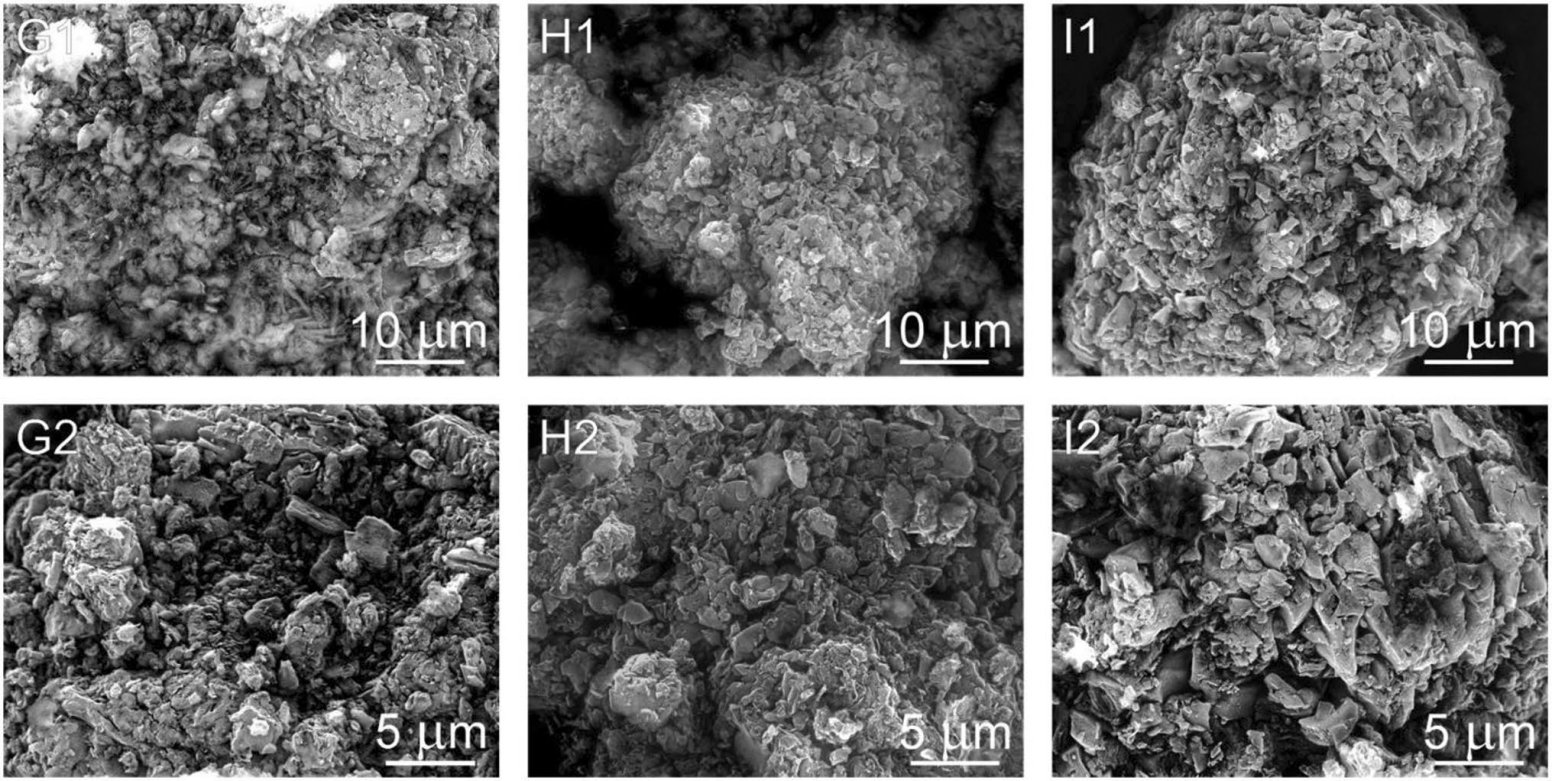
Micrographs (SEM:SE + BSE) of CN-GM 0.5 (**G1,G2**), CN-GM 2 (**H1,H2**) and CN-G 4 (**I1,I2**).

The surface morphology of CN-D, CN-M, CN-G, CN-GD (0.5; 2 and 4) and CN-GM (0.5; 2 and 4) were investigated using SEM. In Fig. 9 the micrographs A (A1,A2) represent the CN-D material, the micrographs B (B1,B2) and C (C1,C2) represent the CN-M and CN-G materials, respectively. One can see various agglomerated particles with irregular shapes and sizes. Plate-like particles can be observed as well. Similarly, the particles of the CN-GD materials are shown in Fig. 10 and the particles of the CN-GM materials are visible in Fig. 11.

**Figure 12:**
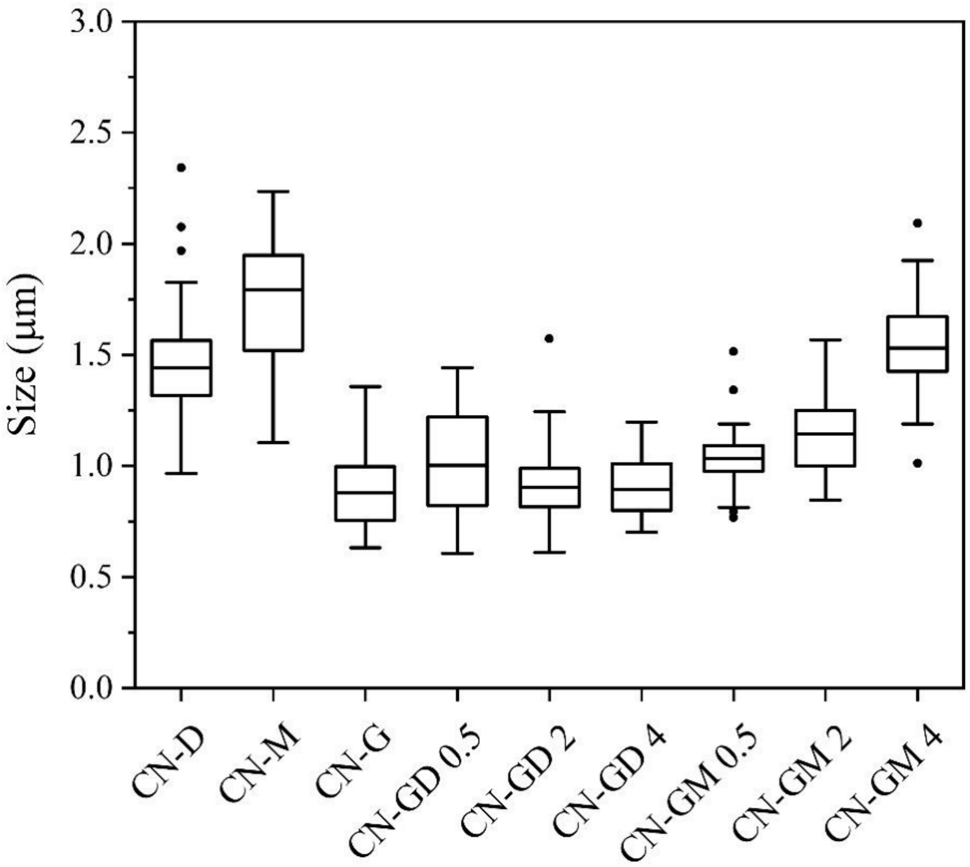
Box plots of sizes of CN materials.

The sizes of CN particles were estimated using the free software Image J. Only completely visible particles located on a surface of bigger agglomerates were measured. The basic statistics are summarized in Table S3 and the size box plots are shown in Fig. 12. The mean sizes were compared with each other by means of their z-scores. The CN-D, CN-M and CN-GM 4 materials had the particles with sizes of 1.49 μm, 1.75 μm and 1.54 μm, respectively. It is obvious in Fig. 12 and was also confirmed by the z-scores that the particles of CN-D (z-score = 0.745), CN-M (z-score = 0.741) and CN-GM 4 (z-score = 0.575) were bigger than those of the other materials whose sizes were similar (Table S3), i.e. 0.89–1.16 μm.

It was also demonstrated that pure guanidine hydrochloride formed smaller particles than pure melamine and dicyandiamide. The materials synthetized from the mixtures of guanidine hydrochloride formed CN particles of a similar size except for CN-GM 4. This material consisted of a much higher content of guanidine hydrochloride than melamine (Table 1). Therefore, guanidine hydrochloride reacted with its other molecules providing melamine, which consequently formed bigger particles in CN-GM 4 similarly as was documented for pure melamine (CN-M).

### DSC and TGA study

The DSC and TGA study were performed on both pure precursors and their mixtures. Differential scanning calorimetry showed the formation of melamine and melamine-like intermediates which were present for the mixtures but not for the pure precursors. This means that the formation of these graphitic carbon nitrides led though different pathways rather than the traditional one^28^, see below.

**Figure 13:**
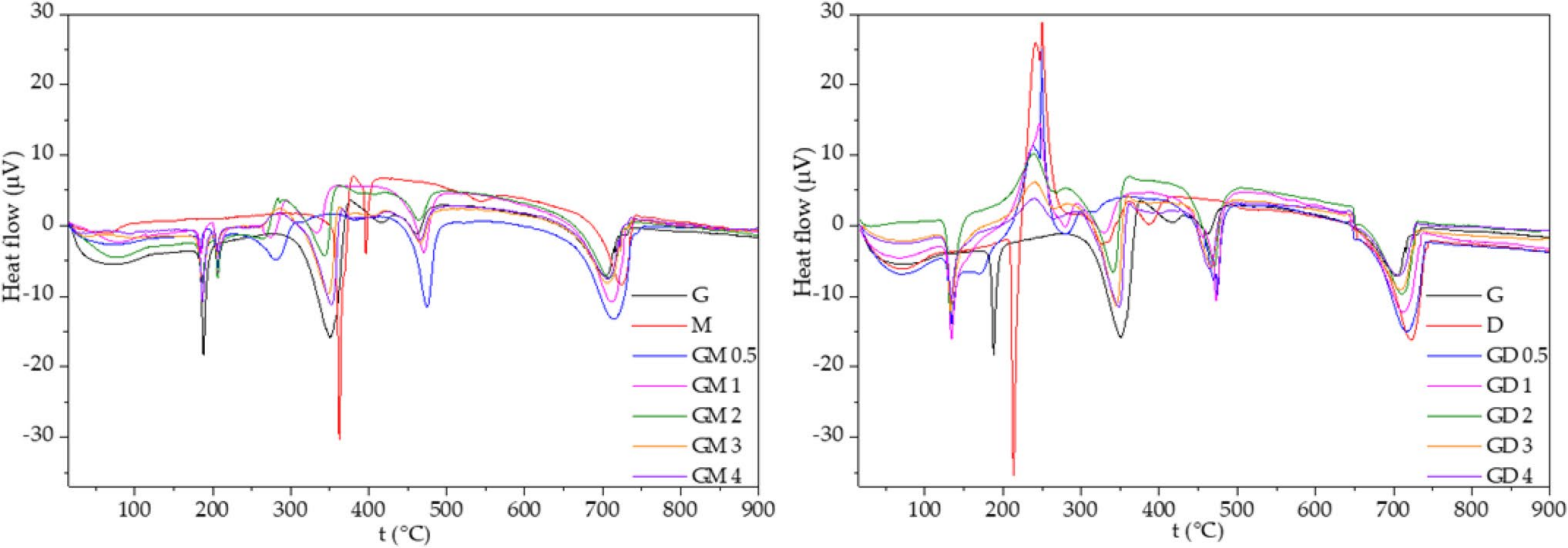
DSC curves of CN materials.

The DCS curves for the GM series shown in Fig. 13 can be divided into five main endothermic peak areas. Guanidine hydrochloride melts clearly at 188 °C (corresponding to previous characterizations^44^); this endothermic peak is slightly shifted in the GM series (186–187 °C). The next endothermic peak, exclusive to the GM series, at 206 °C can be attributed to the melting of guanidyl-triazine (see Fig. 13). The melting of melamine at 334–352 °C was slightly shifted to lower temperatures from the melting point of pure melamine (362 °C^45^) and with significantly broadened peaks. This is clearly due to the interaction between the already melted guanidine-hydrochloride and melamine, and perhaps also as an effect of the presence of guanidine-melamine reaction products (Fig. 13). At the temperatures 464–475 °C the CN-GM material was formed from the GM mixtures. The temperatures of thermal polymerization of the GM mixture lay between the temperatures of polymerization of pure parent compounds guanidine hydrochloride (462 °C) and melamine (544 °C^46^), significantly closer to the first one. The G/M ratio seems to have no effect on the actual value of the temperature of polymerization. These findings indicate that the presence of guanidine hydrochloride lowers the temperature of CN polymerization in comparison with pure melamine. The last area above 700 °C presents a decomposition of the CN-structure with nearly 100% mass loss.

The DSC curve of pure DCDA (Fig. 13) is different from those of the other precursors and their mixtures. The endothermic peak at 214 °C corresponds to the melting of DCDA but the intensive broad exothermic peak at 250 °C is attributed to its polymerization^47^. This exothermic peak is visible in all GD series. The formation of CN was indicated at 391 °C which is lower than those found for guanidine as well as melamine (see above).

**Figure 14:**
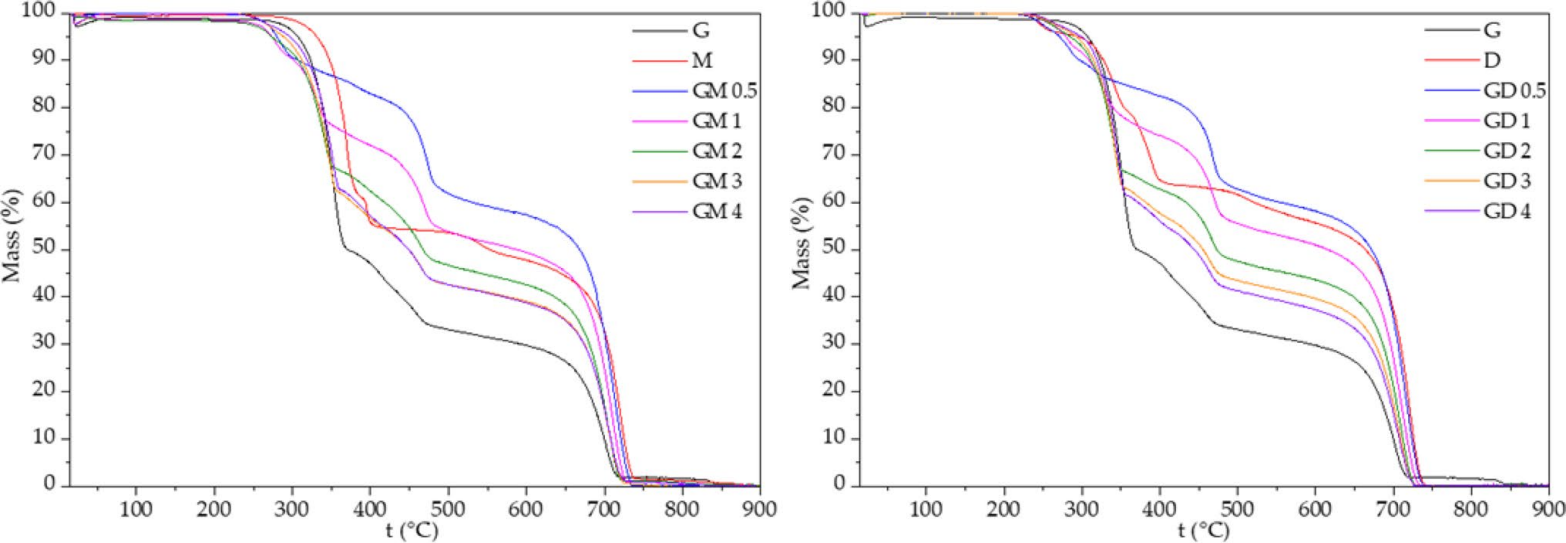
TGA curves of CN materials.

The melting of the GD mixtures was found at 132–134 °C which does not correspond either to the melting point of guanidine hydrochloride (188 °C) or DCDA (214 °C). This is probably caused by the melting of their eutectic mixture. The formation of biguanide and triguanide via the condensation of guanidine hydrochloride and dicyandiamide (Fig. 14) may also play a role in this shift. The next intensive endothermic peaks at 330–348 °C was attributed to the melting/formation of triazine or polyguanide products from DCDA. The formation of CN was observed at 464–474 °C, that is, roughly at the same temperature as in case of the GM mixtures. The decomposition of CN structure was found at temperatures above 700 °C, as in the previous experiments.

From the thermogravimetric measurements shown in Fig. 14 the mass loss due to ammonia release as a result of the gradual formation of melem, melon and finally graphitic carbon nitride is visible^46^. It is also visible that the presence of guanidine hydrochloride in a mixture or as a pure compound accelerates the formation of CN approximately down to 450–475 °C. Moreover, the presence of guanidine hydrochloride in the mixture seems to make the ammonia loss more gradual, and adds more steps in the pathway of CN-formation. Above temperatures of about 650 °C, polymeric structures of graphic carbon nitride start to decompose rapidly.

### Explanation of obtained results and proposed reaction mechanisms

All of the prepared materials CN-G, CN-M, CN-D, CN-GM and CN-GD were found to be similar CN materials in term of chemical composition (Table 4) and structure documented by the XRD patterns and FTIR spectra. Yet their photocatalytic properties, as well as their specific surface area analysed by the physisorption of nitrogen and SEM, differ significantly. To explain these observations one hint is given by the DSC and TGA records (Figs. 13, 14). These suggest that while the resulting materials are similar the pathways to their creation are different in terms of the pure precursor materials (guanidine hydrochloride, melamine, DCDA) and their GM and GD mixtures.

To demonstrate these differences among the SSAs of CN-G, CN-M and CN-D can be taken as an example (Table 2). CN-G has approx. twice the SSA than CN-M and three times more than CN-D (the values are 23, 12 and 8 m^2^ g^−1^, respectively). The formation of one melem core from guanidine presumes an elimination of 8 molecules of NH_3_ and 6 molecules of HCl per one C_6_N_7_ (heptazine) core and the condensation of six guanidine hydrochloride molecules. At the same time, in the case of melamine and DCDA, only 2 molecules of NH_3_ have to be eliminated and the condensation of two melamines and three DCDA molecules are necessary to form one C_6_N_7_ core.

In the case of melamine, a ring-opening and two ring-closure reactions have to be presumed, while in the case of DCDA only three ring-closure reactions may occur. It is safe to assume that with the number of particular steps necessary to form a heptazine core (the condensation of particular heptazines takes similar pathways in all of the three cases) the probability of structure imperfections increases. Additionally, the number of structure imperfections (cavities in the structure, imperfect heptazine condensations, etc.) enlarges the SSA and has an impact on the photocatalytic properties. From the aforementioned it follows that guanidine hydrochloride is important for the increase of SSA by (i) the release of a huge amount of gases, such as NH_3_ and HCl, creating a lot of pores in the CN structure^48^ and (ii) the formation of complex reactions (Figs. S6, S7) resulting in structure imperfections. The same idea can be applied to the properties of the CN-GM and CN-GD materials.

**Figure 15:**
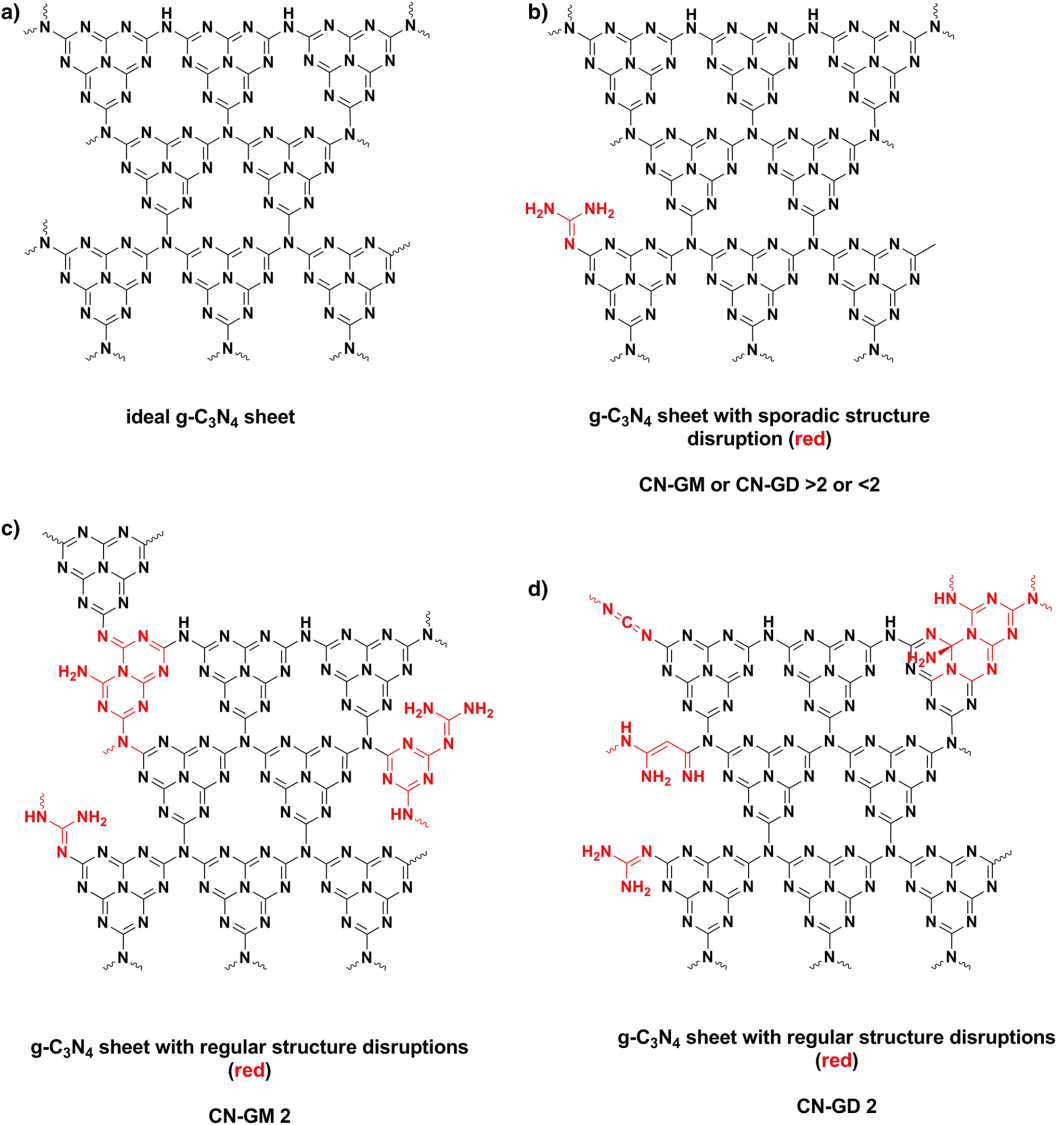
Graphitic carbon nitride sheets with and without structural disruptions (g-C_3_N_4_ stands for CN). (**a**) ideal CN sheet, (**b**) CN-GM or CN-GD sheet with sporadic structural disruption, (**c**) CN-GM 2 sheet with regular structural disruption), (**d**) CN-GD 2 sheet with regular structural disruption. (g-C_3_N_4_ stands for CN).

In the case of the CN-GM series, a ring opening of melamine occurs in the first step according to the ANRORC mechanism^49^. The guanidine in the mixture connects to the “ring-open form” tetraguanide followed by the re-closure of the structure to a guanidyl-triazine. The ring openings, condensation and closings occur until melem units with residual groups (guanide-like) are formed with structural disruptions demonstrated in Fig. 15 in red colour. This route will produce structural imperfections made by the unfinished condensation of heptazine, as triazine- and triazino[1,2-a]triazine units will occur. This explains the increase in cavities and the specific surface area of the CN-GM materials and the enhancement of photocatalytic properties because all of the structure disruptions may result in the formation of catalytically active sites.

In the case of the CN-GD series the condensation of guanidine and dicyandiamide occurs directly without the need of ring-opening due to the absence of melamine in the initial mixture. The polymerization continues via the condensation of triazine or melem intermediates with guanidine-residual groups with oligo-/polyguanides. This leads to another type of structural disruptions, see Fig. 15. An “overcondensation”—an interaction of polyguanide intermediates with the heptazine structure will occur causing also an increase in the SSA and an increase in the photocatalytic ability (proportional to the increase of SSA).The maxima of both photocatalytic properties and SSA can be found for the CN-GM2 and CN-GD2 materials. This means that the stoichiometry leading to the most structural imperfections/terminations lays in a G:M molar ratio of around 1.5:1 and in a G:D molar ratio of around 2:1 (Table 1). In those ratios the presence of guanidine hydrochloride probably alters the reaction routes leading to a CN lattice formation by opening a number of alternative reaction pathways, which is visible also on the DSC/TGA records (Figs. 13, 14). The chemical explanation of possible reactions was given above.

#### Role of chlorine in synthesis of graphitic carbon nitride

Recently, a new strategy for the synthesis of graphitic carbon nitride using nitrogen-rich precursors containing HCl was reported in several papers^48,50–52^. The presence of chlorine in precursor mixtures was shown to alter the formation of CN lattice in favour of its photocatalytic properties. A great deal of the HCl influence is due pre-organization of the precursors into supramolecular assemblies in the reaction mixtures resulting in the final morphology, specific surface area, and catalytic properties^51,52^. While active chlorine usually enters the reactions of precursors and enhances the condensation processes, often ending up in the structure of resulting materials^53^, anionic chloride can intercalate the final CN structure^54^.

The synthesis of the CN-G, CN-GM, and CN-GD materials was based on guanidine hydrochloride, but despite this, chlorine was detected in the final materials neither by XRF nor by FTIR in terms of C–Cl and N–Cl bonds. Furthermore, TGA and DSC spectra did not indicate the hydrochloride cross-formation as well. Some HCl elimination processes occurred during the multiple stages of CN syntheses and likely supported the creation of structural imperfections and disruptions, resulting in increasing the SSA with surface defects beneficial for charge separation^50,51^, which resulted in the enhanced photocatalytic properties.

## Conclusions

Two series of CN materials were synthetized to find the role of guanidine hydrochloride in the synthesis of graphitic carbon nitride. The CN-GM series were synthetized from mixtures of guanidine hydrochloride and melamine and the CN-GD series were synthetized from mixtures of guanidine hydrochloride and dicyandiamide. The CN materials from the pure precursors were synthetized as well.

The band gap energies of the CN materials were changing randomly in the range from 2.67 to 2.73 eV. The XRD and FTIR analyses identified the presence of graphitic carbon nitride, no matter what precursors were used for its synthesis. The SEM revealed that CN prepared from pure guanidine hydrochloride or from its mixtures with other precursors gave rise to the smaller particles of 0.89–1.16 μm in size (1.75 μm for CN-M and 1.49 μm for CN-D) which also corresponds to the decreasing crystallite sizes from 7.50 to 4.27 nm with the increasing content of guanidine hydrochloride in the mixtures. The TGA and DSC analyses explained the temperature behaviour of all the precursors which contributed to an explanation of the reaction pathways of guanidine hydrochloride and melamine/DCDA and the suggestions of the final disruptive CN structure formations.

One material from each series consisting of two mass parts of guanidine hydrochloride and one part of melamine (the GM series) or DCDA (the GD series) possessed the highest specific surface area of 54 m^2^ g^−1^ and 35 m^2^ g^−1^, respectively, and the highest photocatalytic activity concerning the degradation of amoxicillin, Rhodamine B and phenol. The zero-order kinetics of all the CN materials and the organic compounds were observed. Superoxide radicals were found to be the main photocatalytic agents. The photocatalytic stability of the CN materials was proved by the 5 repeated degradations of amoxicillin. The PL decay study revealed that the lifetimes of photoinduced electrons and holes were independent of the CN materials’ composition and these charge carriers recombined with localised luminescence centres. The significant correlations between the photocatalytic reaction rate constants and the SSAs indicated the photocatalytic activities of the CN materials were dependent on their specific surface areas (r = 0.916 for amoxicillin, r = 0.898 for RhB and r = 0.886 for phenol).

The main role of guanidine hydrochloride in the synthesis of graphitic carbon nitride was found in terms of affecting its specific surface areas. The SSA increased due to the creation of pores as a result of the releasing of NH_3_ and HCl and due to the HCl complex reactions forming structure imperfections and disruptions. The obtained results revealed how the photocatalytic properties of graphitic carbon nitride can be changed and employed for the degradation of organic environmental pollutants.

## Supplementary Information

Supplementary Information.
