# Supplementary material for: The role of guanidine hydrochloride in graphitic carbon nitride synthesis
Source: Sci Rep. 2021 Nov 3;11:21600. doi: 10.1038/s41598-021-01009-8 (PMC8566454; doi:10.1038/s41598-021-01009-8)
Supplement: Supplementary file 1 — Supplementary Information. [file 41598_2021_1009_MOESM1_ESM.docx]

**Supplementary materials**

**The role of guanidine hydrochloride in graphitic carbon nitride synthesis**

Aneta Smýkalová^1,2^, Kryštof Foniok^1^, Daniel Cvejn^2,3^, Kamil Macej Górecki^2^ and Petr Praus^1,2,*^

^1^Department of Chemistry VSB-Technical, University of Ostrava, 17. listopadu 15,

708 00 Ostrava-Poruba, Czech Republic

^2^Institute of Environmental Technology, CEET, VSB-Technical, University of Ostrava, 17. listopadu 15, 708 00 Ostrava-Poruba, Czech Republic

^3^ENET Centre, CEET, VSB-Technical, University of Ostrava, 17. listopadu 15,

708 00 Ostrava-Poruba, Czech Republic

^*^Correspondence: petr.praus@vsb.cz


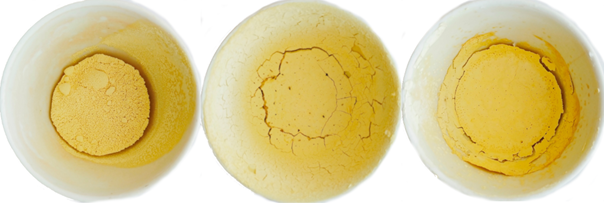


Figure S1. Graphitic carbon nitride prepared from melamine (left), dicyandiamide (middle) and guanidine hydrochloride (right).

Table S1 Χ^2^ values of fitting PL decay curves with various models

| Material | 1^st^ order (Χ^2^) | 2^nd^ order (Χ^2^) | Stretched (Χ^2^) | 3^rd^ order (Χ^2^) |
| --- | --- | --- | --- | --- |
| G | 31.87 | 2.658 | 4.202 | 1.173 |
| M | 27.71 | 2.361 | 4.582 | 1.243 |
| D | 29.35 | 2.647 | 3.225 | 1.105 |
| GM 0.5 | 32.19 | 2.293 | 5.016 | 1.177 |
| GM 1 | 30.34 | 2.533 | 4.035 | 1.192 |
| GM 2 | 31.01 | 2.456 | 3.995 | 1.167 |
| GM 3 | 34.36 | 2.699 | 4.290 | 1.253 |
| GM 4 | 31.38 | 2.472 | 4.143 | 1.063 |
| GD 0.5 | 28.135 | 2.696 | 2.982 | 1.126 |
| GD 1 | 29.72 | 2.307 | 3.832 | 1.120 |
| GD 2 | 31.58 | 2.435 | 4.286 | 1.127 |
| GD 3 | 33.48 | 2.448 | 3.834 | 1.060 |
| GD 4 | 32.47 | 2.456 | 4.408 | 1.180 |

Table S2 B_i_ and τ_i_ coefficients of 3^rd^ order exponential model

| Material | B_1_ | τ_1_ (ns) | B_2_ | τ_2_ (ns) | B_3_ | τ_3_ (ns) |
| --- | --- | --- | --- | --- | --- | --- |
| G | 7529 | 1.23 | 3204 | 4.46 | 530 | 21.45 |
| M | 7331 | 1.35 | 3462 | 4.31 | 534 | 19.47 |
| D | 6877 | 1.13 | 3489 | 4.22 | 552 | 20.10 |
| GM 0.5 | 7946 | 1.40 | 2816 | 4.81 | 528 | 22.26 |
| GM 1 | 7240 | 1.34 | 3319 | 4.70 | 529 | 21.83 |
| GM 2 | 6789 | 1.33 | 3485 | 4.60 | 621 | 22.37 |
| GM 3 | 7165 | 1.20 | 3506 | 4.27 | 672 | 20.48 |
| GM 4 | 7220 | 1.27 | 3374 | 4.40 | 557 | 21.14 |
| GD 0.5 | 6501 | 1.10 | 3599 | 4.16 | 558 | 20.17 |
| GD 1 | 6588 | 1.27 | 3691 | 4.23 | 651 | 19.96 |
| GD 2 | 7266 | 1.27 | 3455 | 4.38 | 619 | 20.85 |
| GD 3 | 7072 | 1.25 | 3376 | 4.47 | 656 | 21.30 |
| GD 4 | 7345 | 1.33 | 3428 | 4.57 | 634 | 21.02 |

Table S3 Basic statistics of CN particles ( n = 30)

| Materials | Mean | St. dev. mean | Min | Max | Median | St. dev. median | Quartil 1 | Quartil 3 | z-score |
| --- | --- | --- | --- | --- | --- | --- | --- | --- | --- |
| CN-D | 1.49 | 0.30 | 0.97 | 2.34 | 1.44 | 0.05 | 1.32 | 1.56 | 0.745 |
| CN-M | 1.75 | 0.30 | 1.11 | 2.24 | 1.79 | 0.06 | 1.54 | 1.95 | 0.741 |
| CN-G | 0.89 | 0.17 | 0.63 | 1.36 | 0.88 | 0.03 | 0.76 | 0.99 | -0.431 |
| CN-GD 0.5 | 1.02 | 0.25 | 0.61 | 1.44 | 1.00 | 0.09 | 0.82 | 1.22 | -0.610 |
| CN-GD 2 | 0.93 | 0.19 | 0.61 | 1.57 | 0.90 | 0.04 | 0.82 | 0.99 | -0.481 |
| CN-GD 4 | 0.90 | 0.14 | 0.70 | 1.20 | 0.89 | 0.04 | 0.80 | 1.01 | -0.336 |
| CN-GM 0.5 | 1.04 | 0.15 | 0.77 | 1.51 | 1.03 | 0.02 | 0.98 | 1.09 | -0.363 |
| CN-GM 2 | 1.16 | 0.20 | 0.85 | 1.57 | 1.14 | 0.05 | 1.01 | 1.25 | -0.488 |
| CN-GM 4 | 1.54 | 0.23 | 1.01 | 2.09 | 1.53 | 0.05 | 1.43 | 1.67 | 0.578 |

Note: All units are micrometres.


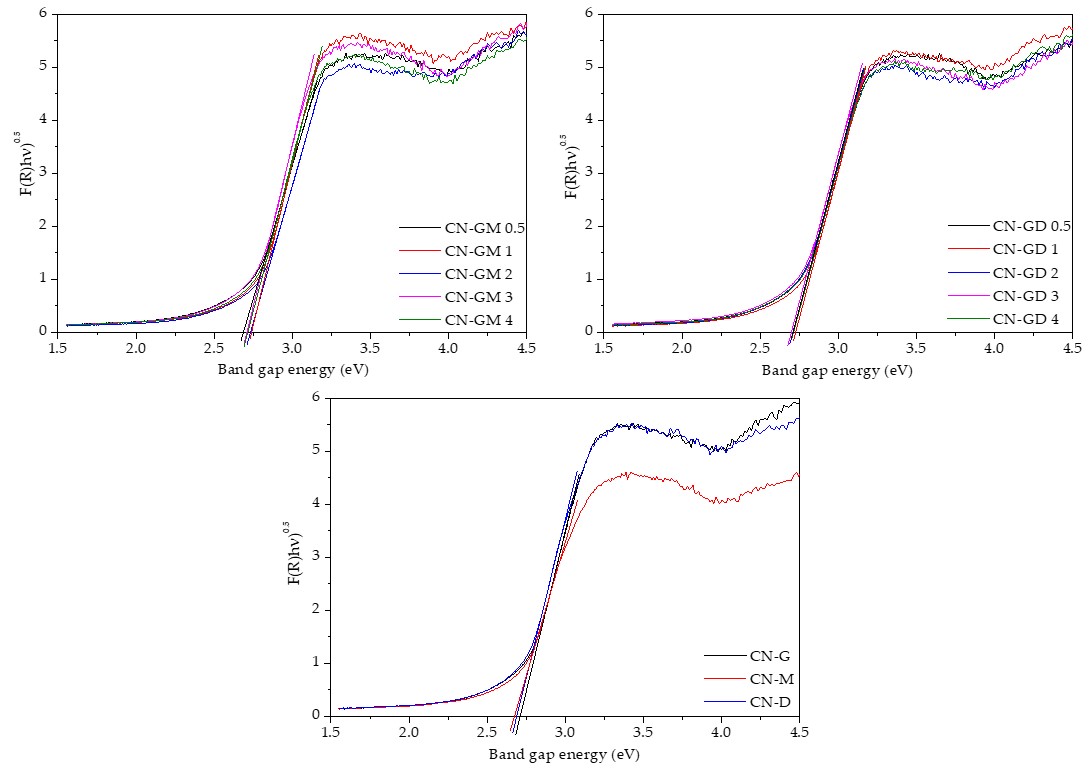


Figure S2 Tauc plots of CN materials.


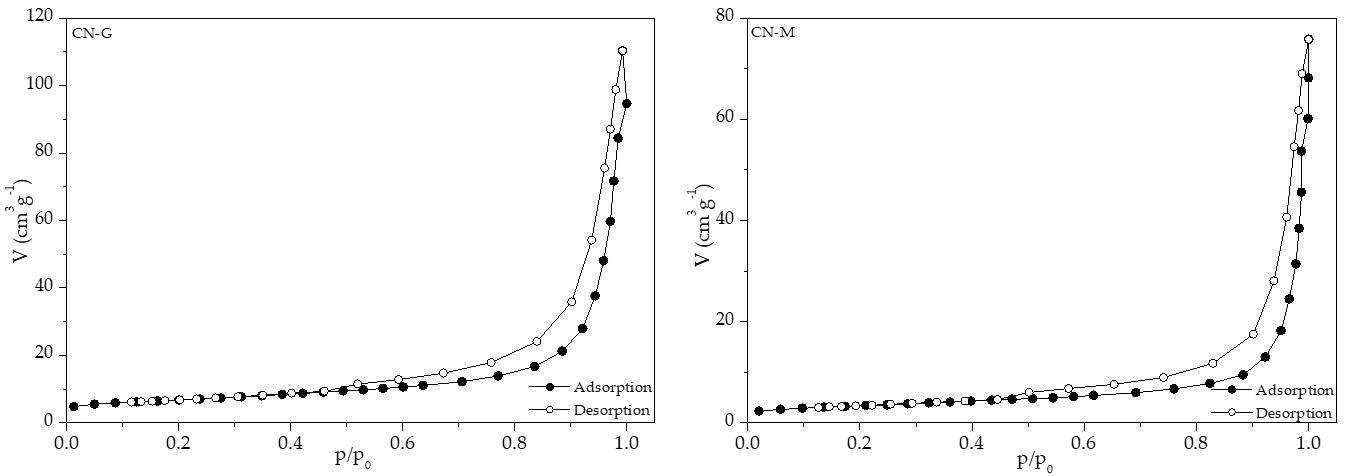


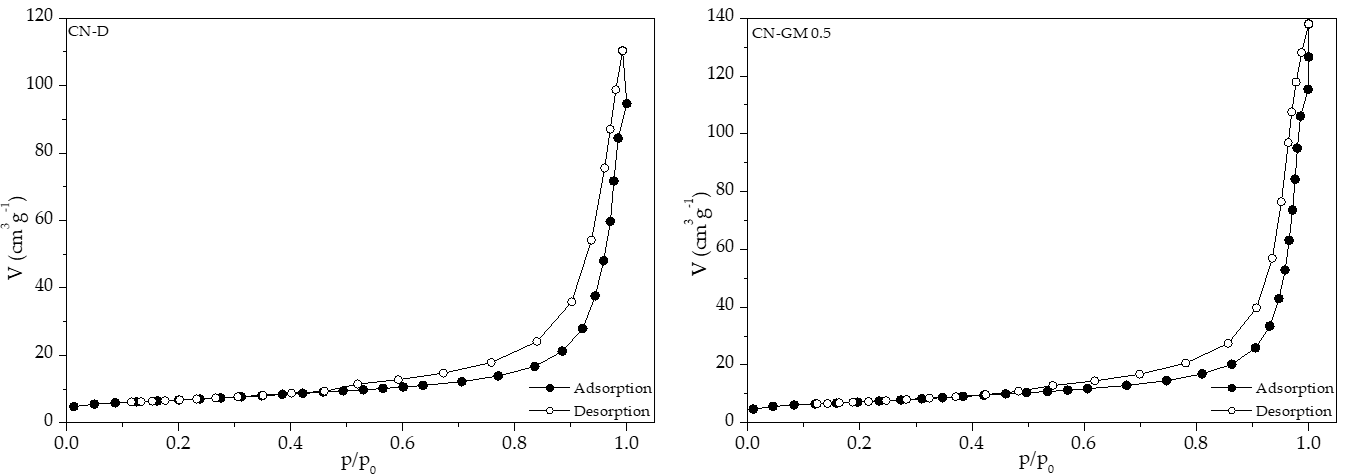


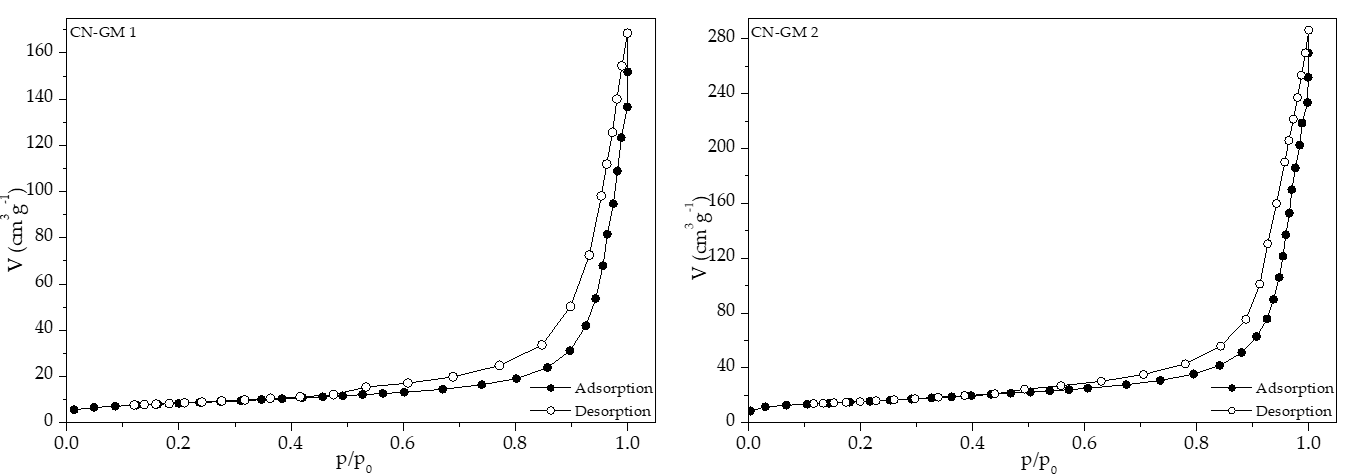


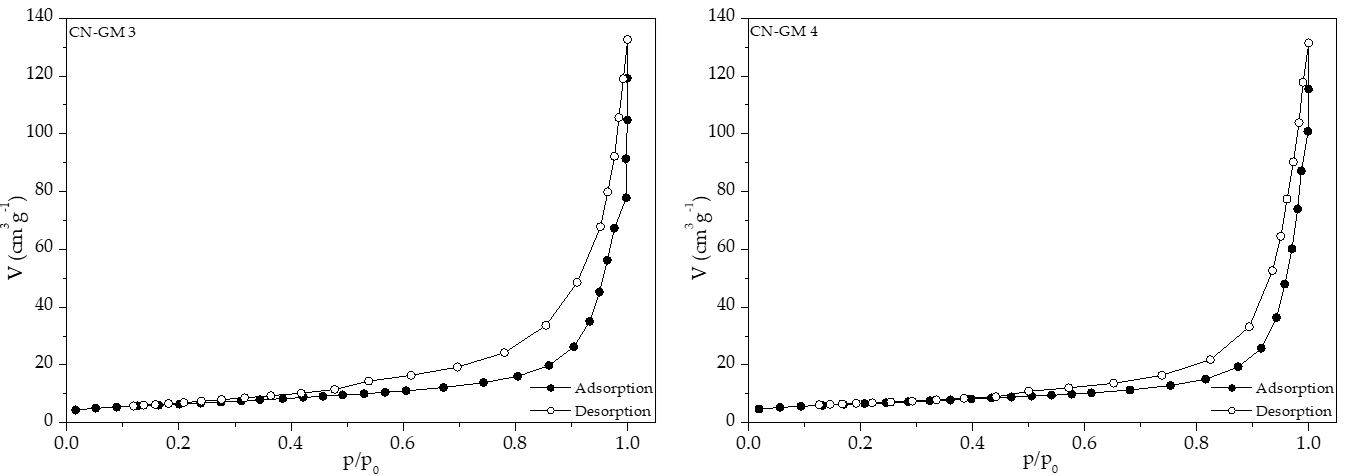


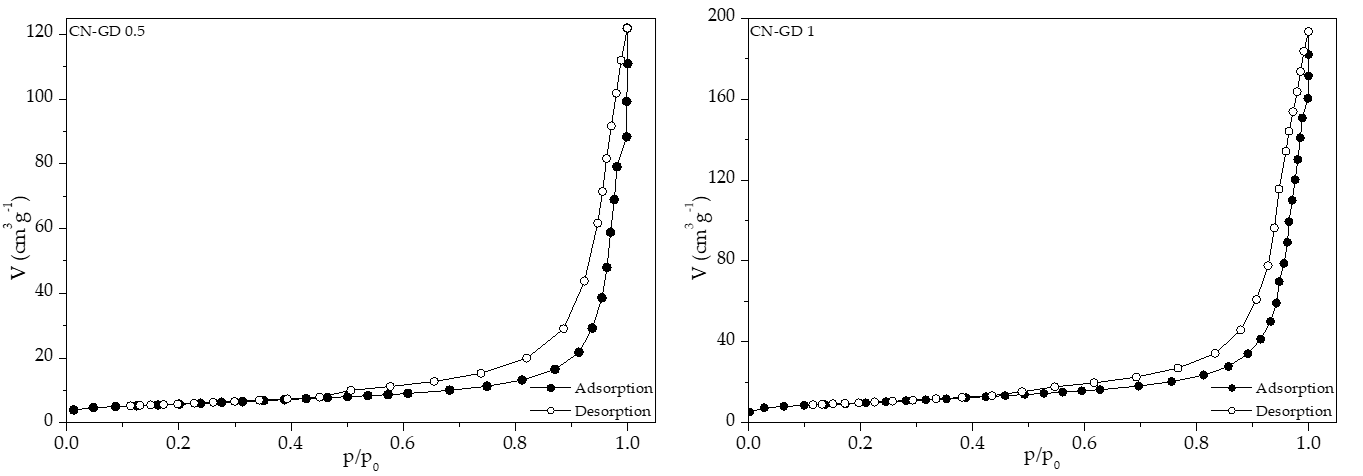


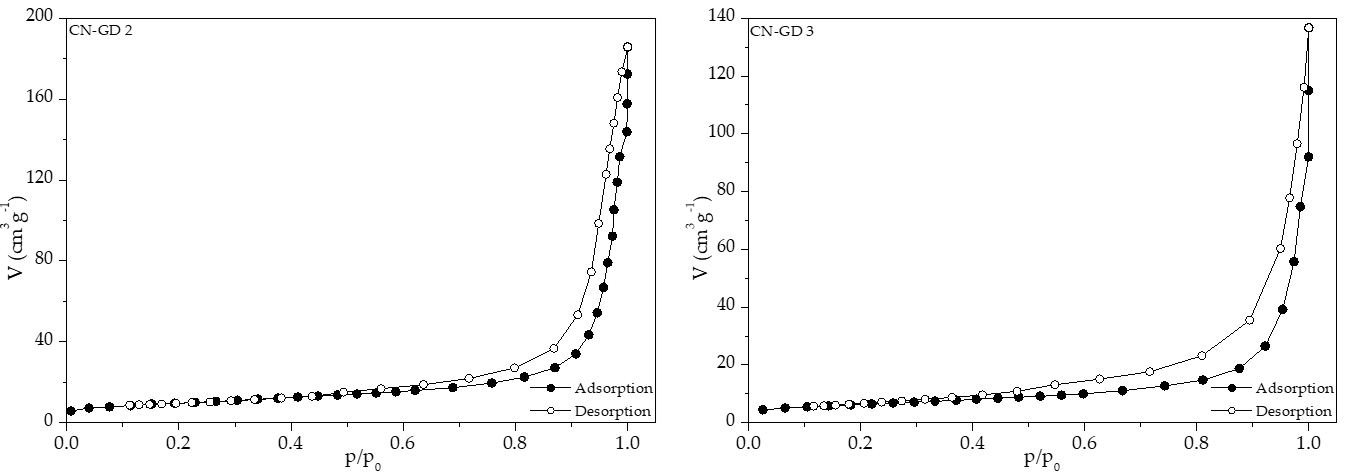

Figure S3. Adsorption-desorption isotherms of nitrogen at 77 K.

**
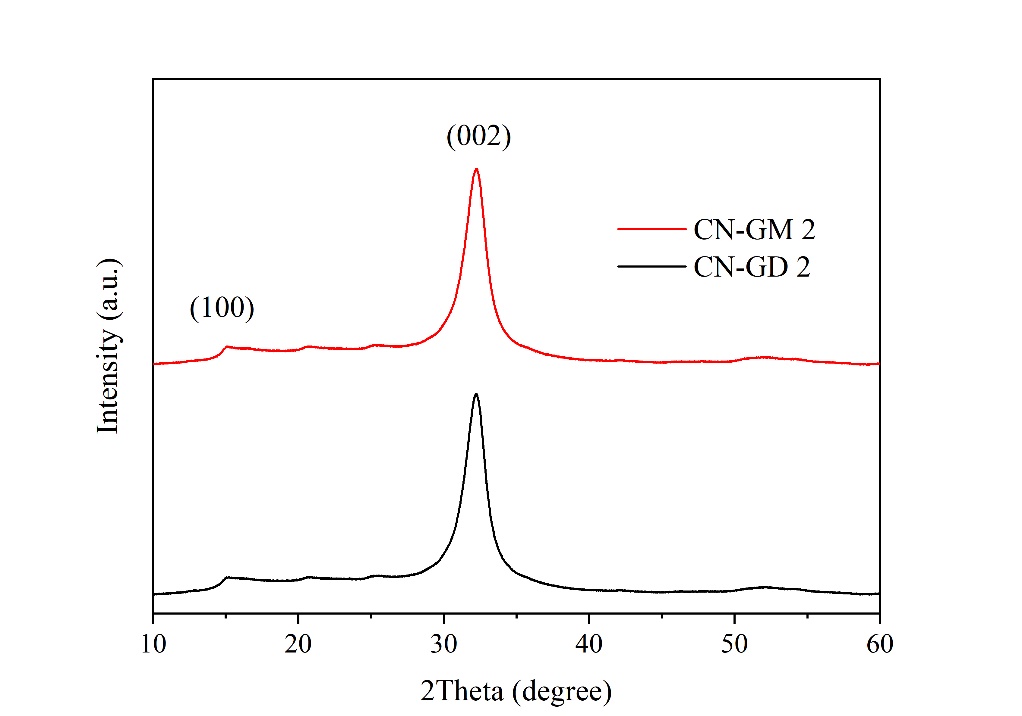
**

Figure S4 XRD patterns of CN-GM 2 and CN-GD 2 after fifth cycle.

**
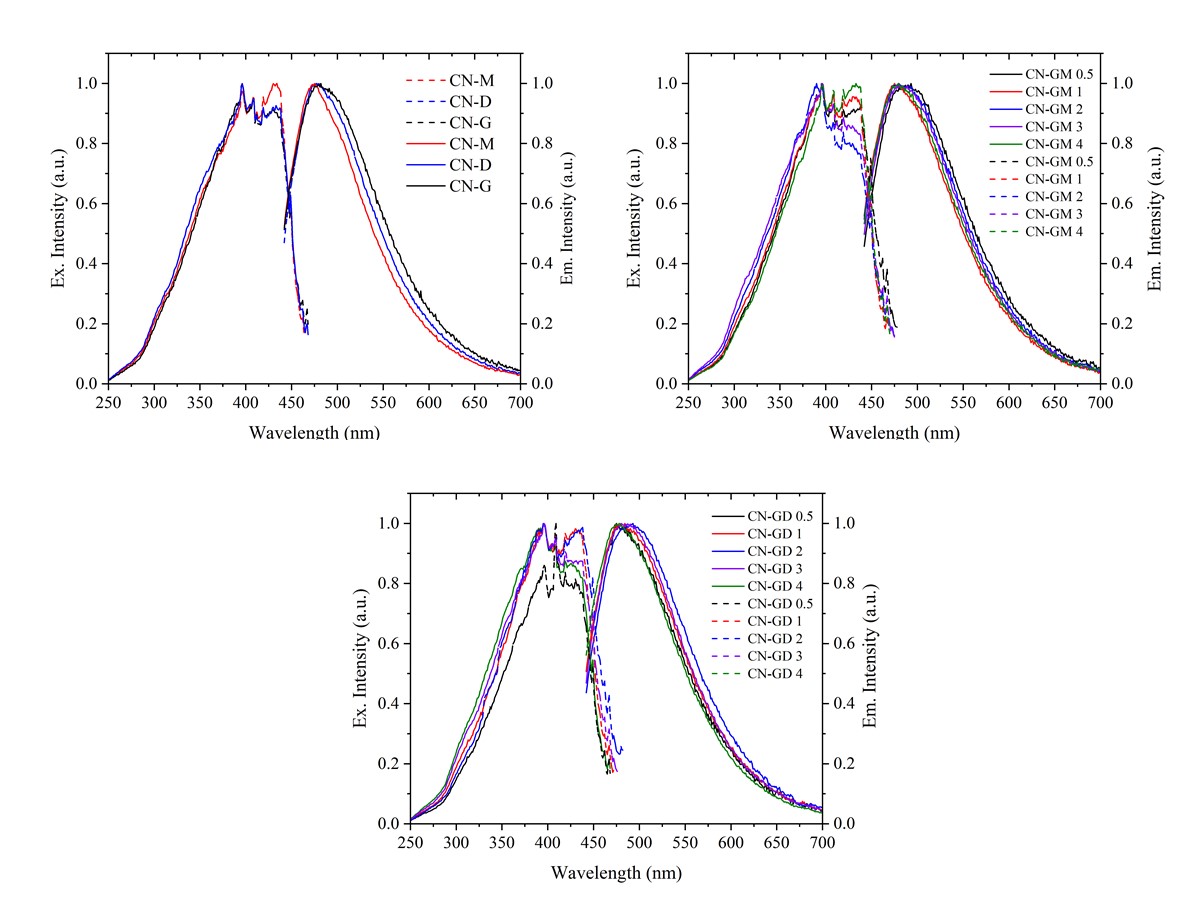
**

Figure S5 Normalized excitation and emission photoluminescence spectra of CN materials.

**
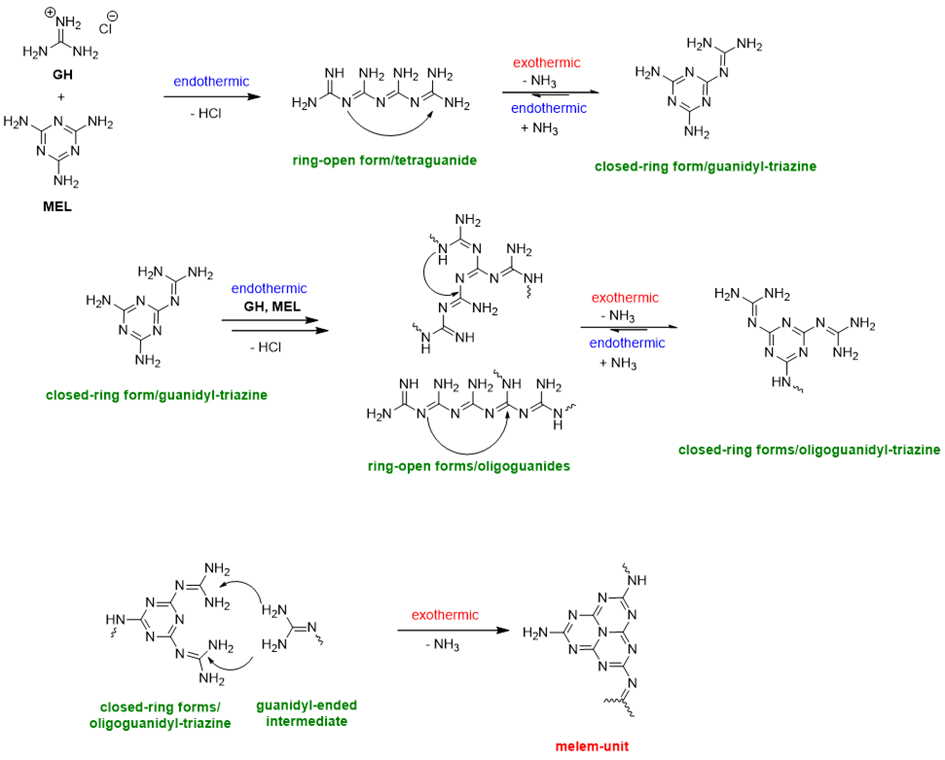
**

Figure S6. Route of CN from guanidine hydrochloride and melamine. GH – guanidine hydrochloride, MEL – melamine.

**
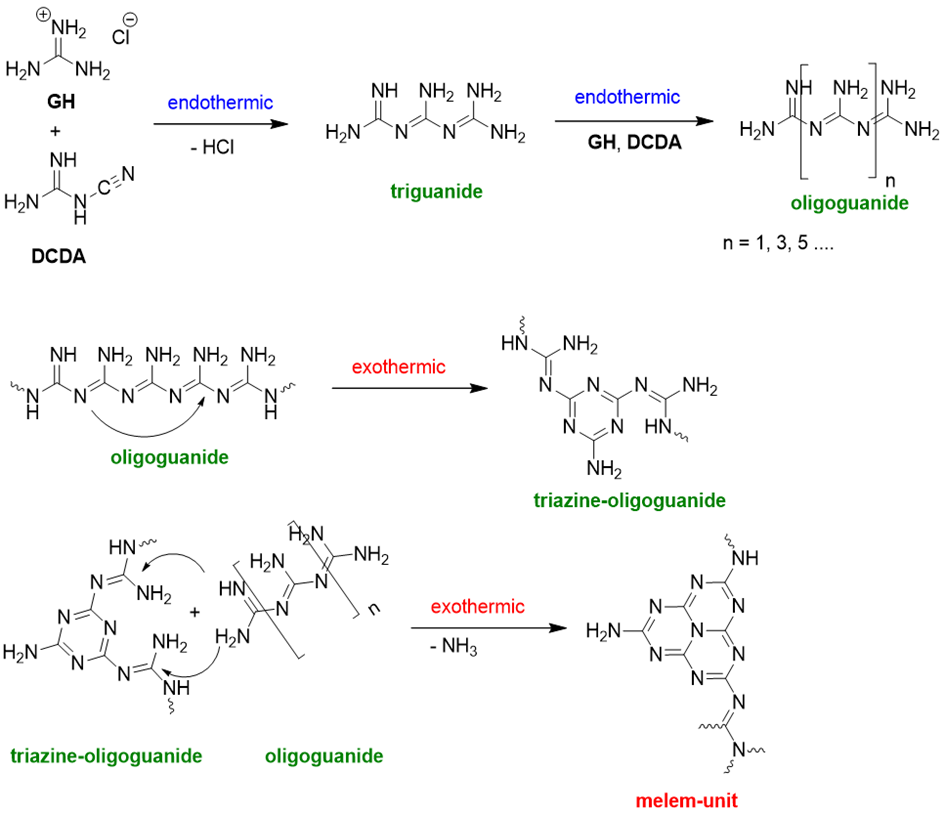
**

Figure S7. Route of CN synthesis from guanidine hydrochloride and dicyandiamide. GH – guanidine hydrochloride, DCDA – dicyandiamide.
